# Binding studies of a putative *C*. *pseudotuberculosis* target protein from Vitamin B_12_ Metabolism

**DOI:** 10.1038/s41598-019-42935-y

**Published:** 2019-04-23

**Authors:** Rafaela dos S. Peinado, Danilo S. Olivier, Raphael J. Eberle, Fabio R. de Moraes, Marcos S. Amaral, Raghuvir K. Arni, Monika A. Coronado

**Affiliations:** 1 Multiuser Center for Biomolecular Innovation, Departament of Physics, Instituto de Biociências Letras e Ciências Exatas (Ibilce), Universidade Estadual Paulista (UNESP), São Jose do Rio Preto-SP, 15054-000 Brazil; 2 Institute of Physics, Federal University of Mato Grosso do Sul, Campo Grande, MS 79090-700 Brazil

**Keywords:** Computational biophysics, Solution-state NMR

## Abstract

Vitamin B_12_ acts as a cofactor for various metabolic reactions important in living organisms. The Vitamin B_12_ biosynthesis is restricted to prokaryotes, which means, all eukaryotic organisms must acquire this molecule through diet. This study presents the investigation of Vitamin B_12_ metabolism and the characterization of precorrin-4 C(11)-methyltransferase (CobM), an enzyme involved in the biosynthesis of Vitamin B_12_ in *Corynebacterium pseudotuberculosis*. The analysis of the *C*. *pseudotuberculosis* genome identified two Vitamin B_12_-dependent pathways, which can be strongly affected by a disrupted vitamin metabolism. Molecular dynamics, circular dichroism, and NMR-STD experiments identified regions in CobM that undergo conformational changes after s-adenosyl-L-methionine binding to promote the interaction of precorrin-4, a Vitamin B_12_ precursor. The binding of s-adenosyl-L-methionine was examined along with the competitive binding of adenine, dATP, and suramin. Based on fluorescence spectroscopy experiments the dissociation constant for the four ligands and the target protein could be determined; SAM (1.4 ± 0.7 µM), adenine (17.8 ± 1.5 µM), dATP (15.8 ± 2.0 µM), and Suramin (6.3 ± 1.1 µM). The results provide rich information for future investigations of potential drug targets within the *C*. *pseudotuberculosis’s* Vitamin B12 metabolism and related pathways to reduce the pathogen’s virulence in its hosts.

## Introduction

Vitamin B12 (cobalamin), the so called ‘antipernicious anaemia factor’ was discovered by Minot and Murphy in 1926 and it was purified from liver and kidney in 1948. The vitamin is largely present in two biological forms: adenosylcobalamin or methylcobalamin^1^. Biologically, Vitamin B_12_ participates as a coenzyme in various metabolic processes, such as the complex rearrangements and reductions through methylation^2^. It also functions as a cofactor of methyltranferases and is necessary for methionine synthesis, methanogenesis, CO_2_ fixation, and even in the regulation of bacterial gene expression, where it functions as a ligand of mRNA elements (riboswitches) for genetic control^2,3^. Since Vitamin B_12_ biosynthesis is confined to prokaryotes, all eukaryotic organisms must acquire Vitamin B_12_ through diet.

**Figure 1:**
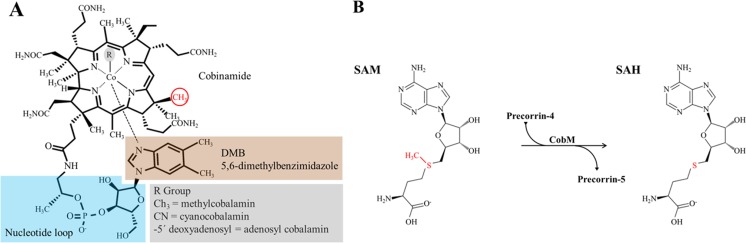
Molecular structure of Vitamin B_12_ and reaction mechanism of CH_3_-group transfer by the precorrin-4 C(11)-methyltransferase (CobM). (**A**) Structure of Vitamin B_12_ and B_12_-derived cofactors. In red, the methyl group transferred by CobM. (**B**) Reaction mechanism of CobM, the cofactor is SAM which donate the methyl group (in red) for the transfer reaction.

Vitamin B_12_ has one of the most complex structures of any of the biological cofactors, containing a tetrapyrrole framework with a centrally chelated cobalt ion held in place by a lower axial base, dimethylbenzimidazole (DMB), and an upper ligand (R group) determines the B_12_ form^4^ (Fig. 1A).

The R group contains a cyano group (Cn-Cbl), deoxyadenosine group (Ado-Cbl) or a methyl group (Met-Cbl)^4,5^. The molecular structure is very complex and its biosynthesis requires around 30 enzyme-mediated steps^6–8^. The biosynthesis of Vitamin B_12_ is divided into three main processes: the synthesis of the corrin ring, the construction of the lower axial ligand, and finally the reunion of the components to yield the final coenzyme. There are two distinct pathways^9^ of Vitamin B_12_ biosynthesis, the aerobic and anaerobic. The enzymes that participate in the aerobic pathway contain the prefix Cob, whereas those of the anaerobic pathway are termed Cbi.

The addition of eight S-adenosyl-L-methionine (SAM) methyl groups to the tetrapyrrole structure during the first part of Vitamin B_12_ biosynthesis is performed by six transmethylases, which are highly related in structure and in function. The crystal structures of five transmethylases are shown in supplementary Fig. S1. However, besides the strong relation regarding the protein structure, a sequence alignment of the six transmethylases of *C*. *pseudotuberculosis* involved in Vitamin B_12_ synthesis demonstrated strong variations (Supplementary Fig. S2). The glycine-rich sequence GAGPGD, which is involved in SAM/SAH binding^10^, is conserved. Based on bioinformatics analysis of these six transmethylases of *C*. *pseudotuberculosis*, Precorrin-4 C(11)-methyltransferase (CobM) was chosen to perform the subsequent study.

CobM functions as a transmethylase in the oxygen-dependent pathway, similar to its homologue CbiF. CobM/CbiF methylates C11 of precorrin-4 during the aerobic biosynthesis of Vitamin B_12_, generating precorrin-5^10^ (Fig. 1B).

Investigation of any of the six transmethylases offers insights into the mechanisms of precorrin methylation and additionally provides a model on which to base the catalytic process of the other precorrin methylases. As described above, the SAM binding area of the six *C*. *pseudotuberculosis* transmethylases is highly conserved, the identification of a molecule, which competes for the same binding area of one transmethylase, may also effect also the other proteins of this family. It has already been reported that transmethylases are essential in the Vitamin B_12_ biosynthesis in the aerobic pathway CobA^11,12^, and CbiD and G in the anaerobic pathway^13^.

*Corynebacterium pseudotuberculosis* is a gram-positive facultative intracellular pathogen and the agent of caseous lymphadenitis (CLA) in equids, sheep, goats, and to a lesser extent in horses and cattle. This disease can lead to considerable economic loss in many countries, including Brazil, for it affects the yields in wool and milk production, animal weight loss resulting in carcass condemnation and it often results in death due to the formation of abscesses in the visceral and superficial lymph nodes, as well as, caseous necrosis of lymphatic glands^14,15^. So far, no effective treatment for the disease is available.

*C*. *pseudotuberculosis* is a member of the heterogeneous CMNR-group of pathogens, which forms a cluster of Gram-positive bacteria along with *Mycobacterium*, *Nocardia*, and *Rhodococcus* genus^15^. The Vitamin B_12_ metabolic pathway has been considered a possible target for the development of drugs in *M*. *tuberculosis*^4^ a closely related organism of *C*. *pseudotuberculosis*.

Analysis of the *C*. *pseudotuberculosis* genome identified two B_12_-dependent enzymes and pathways. However, little is known about Vitamin B_12_ metabolism in *C*. *pseudotuberculosis* and in the B_12_-dependent pathways. In this context, our study describes the genome analyses of *C*. *pseudotuberculosis* strain 1002 (bv. ovis) and *C*. *pseudotuberculosis* strain CIP52.97 (bv. equi) regarding enzymes involved in Vitamin B_12_ synthesis and the identification of Vitamin B_12_-dependent enzymes/pathways. A protocol for the large scale production of *Cp*-CobM in *E*. *coli* was developed. So far, no crystal structure of *Cp*-CobM was solved (or any other *C*. *pseudotuberculosis* protein involved in the vitamin B_12_ synthesis), therefore, homology modeling with a homologue crystal structure of *R*. *capsulatus* CobM (PDB: 3NEI, sequence identity of 51%) was performed. Subsequent docking and molecular dynamics were utilized to understand the conformational changes upon binding of ligands like s-adenosyl-L-methionine (SAM), s-adenosyl-L- homocysteine (SAH). The interaction of SAM was further investigated by NMR-STD experiments as well as the competition studies of adenine, dATP, and suramin for the same binding site. The dissociation constants between *Cp*-CobM and SAM, adenine, dATP, and suramin were determined using fluorescence spectroscopy.

## Results and Discussion

### Proteins participating in Vitamin B_12_ synthesis and Vitamin B_12_-dependent proteins in *C*. *pseudotuberculosis*

The dependence of different enzymes on specific Vitamin B_12_ forms requires that organisms, who utilize B_12,_ possess the enzymatic machinery to synthesize this vitamin. The complete genome of *C*. *pseudotuberculosis* has been deposited in the GenBank-NCBI database (http://www.ncbi.nlm.nih.gov/genbank/)^16,17^. *C*. *pseudotuberculosis* is classified into two biovars: ovis and equi^18,19^. Search in the GenBank-NCBI database revealed that all genes, with the exception of one (CobE), involved in the aerobic Vitamin B_12_ synthesis pathway are conserved in *C*. *pseudotuberculosis* strain 1002 (bv. ovis) and strain CIP52.97 (bv. equi) (Supplementary Table. S1). The arrangements of Vitamin B_12_ genes in the genome of *C*. *pseudotuberculosis* indicate that the differences between biovar ovis and equi are minimal (Supplementary Fig. S3).

The *C*. *pseudotuberculosis* genome contains two genes, which require Vitamin B_12_ as cofactors for activity, Methylmalonyl CoA mutase and Methionine synthase (Table 1).

**Table 1:** Vitamin B_12_-dependent Enzymes present in different bacterium species.

Gene name	Organism/enzyme/reaction/pathway	*Corynebacterium* species
***mutAB***	*M*. *tuberculosis* Methylmalonyl CoA mutase (R)-Methylmalonyl-CoA → Succinyl-CoA Methylmalonate pathway	***C***. ***pseudotuberculosis*** (ADL10538)
***meth***	*M*. *tuberculosis* Methionine synthase 5-Methyltetrahydrofolate + L-homocysteine → Tetrahydrofolate + L-methionine L-methionine biosynthesis	***C***. ***pseudotuberculosis*** (ADL10596)
***nrdZ***	*M*. *tuberculosis* Vitamin B_12_-dependent ribonucleoside-diphosphate reductase 2’-Deoxyribonucleoside diphosphate + Thioredoxin (ox) + H_2_O ↔ Ribonucleoside diphosphate + Thioredoxin (red) DNA replication	*C*. *nephridii*
***eutC***	*M*. *tuberculosis* Ethanolamine ammonia-lyase Ethanolamine → Acetaldehyde + NH_3_ Ethanolamine degradation pathway	*C*. *kroppenstedtii* *C*. *equi* *C*. *lactis RW2-5* *C*. *vitaeruminis DSM 20294*
***btuC***	*N*. *seriolae* Vitamin B_12_ import system permease protein BtuC	*C*. *oculi* *C*. *lowii* *C*. *diphtheriae*

Methylmalonyl CoA mutase requires Ado-Cbl as a cofactor and catalyzes the final step in the methylmalonyl pathway, the reversible isomerization of *R*-methyl-malonyl-CoA to succinyl CoA, by doing so; it fulfills a critical function in modulating intracellular pools of propionyl-CoA, a toxic product of the catabolism of odd- and branched-chain fatty acids and cholesterol^20^. Methionine synthase uses the cofactor Met-Cbl to catalyze the conversion of homocysteine to the essential amino acid methionine^21^. Many bacteria like *M*. *tuberculosis* or *E*. *coli* possess a Vitamin B_12_-independent methionine synthase, MetE (5-methyltetrahydropteroyltriglutamate-homocysteine methyltransferase), which they employ for the same methyltransferase reaction and that represents a backup system for the Vitamin B_12_-dependent system^22^. The MetE gene can be located in the genome of several *Corynebacterium* species, e.g. *C*. *glutamicum*, *C*. *efficiens*, but not in *C*. *pseudotuberculosis* genomes deposited in the GenBank-NCBI database.

The Vitamin B_12_-dependent proteins such as the Vitamin B_12_-dependent ribonucleoside-diphosphate reductase, ethanolamine ammonia-lyase, and Vitamin B_12_ import system permease protein BtuC are not conserved in the *C*. *pseudotuberculosis* genome, but are conserved in the genome of other *Corynebacterium* species.

### Enzyme production and purification

*Cp*-CobM has a molecular weight of 28.974 kDa (272 amino acids). After two step purification, the purity of the protein was confirmed on a denaturating SDS-PAGE (Supplementary Fig. S4A). Dimer conformation of the protein was confirmed by size exclusion chromatography (Supplementary Figs S4B, S5).

### Investigation of the protein secondary structure under the influence of ligands

**Figure 2:**
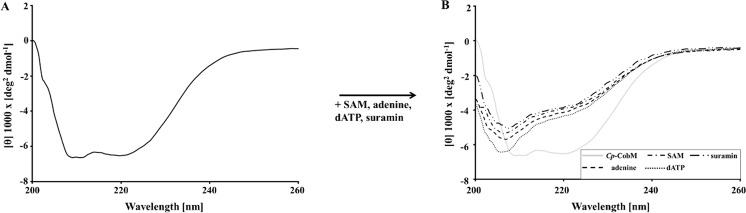
Far-UV CD-spectra from 200 to 260 nm of *Cp*-CobM under different conditions. (**A**) Far-UV CD-spectra of *Cp*-CobM. (**B**) Influence of SAM, dATP, adenine and suramin on the CD-spectra of *Cp*-CobM.

The far-UV CD spectrum of *Cp*-CobM is characterized by the presence of two relative minima at 208 and 220 nm (Fig. 2A) indicating a higher percentage of helical content than β-strand in the protein secondary structure.

The effect of SAM, adenine, dATP, and suramin on the protein secondary structure was tested, indicating a change in the protein secondary structure composition (Fig. 2B).

### Binding studies between *Cp*-CobM and SAM, dATP, adenine, and suramin using Nuclear Magnetic Resonance (NMR)

**Figure 3:**
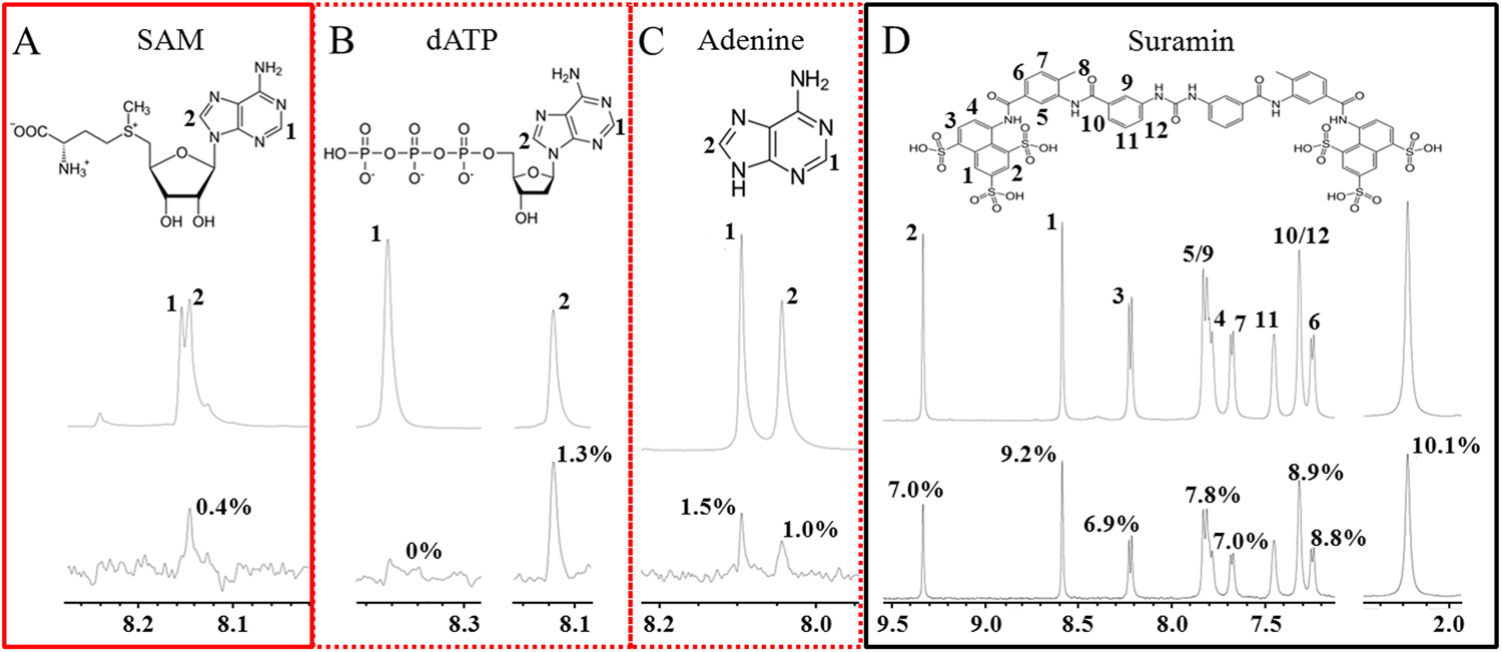
Illustration of the STD-NMR results for *Cp*-CobM with SAM, dATP, adenine and suramin. NMR spectra and binding epitopes are shown in chemical structures; (**A**) STD-NMR results for *Cp*-CobM with SAM. (**B**) STD-NMR results for *Cp*-CobM with dATP. (**C**) STD-NMR results for *Cp*-CobM with adenine. (**D**) STD-NMR results for *Cp*-CobM with suramin. Red box label the native cofactor SAM, dotted red box label the significant competitors (dATP and adenine) for the *Cp*-CobM SAM binding site identified by NMR competition experiments. Black box label suramin, affecting the binding of SAM, but show low competition with SAM.

STD-NMR experiments revealed that SAM, dATP, adenine, and the polyanion suramin interact specifically with *Cp*-CobM. Off-resonance and difference spectra were observed for each ligand independently. SAM has a distinct signal observed in the STD difference spectra, at δ8.14 ppm (Fig. 3A). Calculating the relative ratio between off-resonance and difference spectrum signal area is possible to assess the binding epitopes and, thus, the degree of proximity between ligand atoms and the protein binding site. The closer the ligand atom is to the protein, the more saturation it will receive and, as a result, the higher its signal intensity in the difference spectrum. Binding epitopes for SAM are located at the adenine section of the SAM molecule. Accordingly, dATP and adenine were tested by STD NMR and the binding epitopes were located in the same area, when compared with SAM (Fig. 3B,C). For SAM and dATP, hydrogen bound to carbon 2 is seen in the difference spectrum, while other atom signals are not present. Signals from the ribose hydrogens are not observed. On the other hand, for adenine, the highest STD-AF was seen for hydrogen bound to carbon 8 (atom #1 in Fig. 3C) and a hydrogen bound to carbon 2 signal is present. Binding epitopes for suramin were found in the naphthalene groups and the aromatic rings of the molecule (Fig. 3D).

However, the binding epitopes indicate differences; the dATP signal observed in the STD difference spectra at δ8.33 ppm (Fig. 3B), has no significant magnetization transfer. These differences can explain the role of the interactions of the methionine (SAM) or phosphate groups (dATP). The methionine molecule of SAM interacts with CobM proteins as demonstrated in Fig. 3A.

Schubert *et al*. 1998, described the binding of a phosphate ion in *B*. *megaterium*-Cbif SAM binding site^10^, this phosphate ion interacts with His100 (His79 in *Cp*-CobM) and the backbone of Thr101 (Ser80 in *Cp*-CobM). The similarity of *B*. *megaterium*-Cbif in this region (Supplementary Fig. S6) makes the binding of the phosphate group of dATP possible.

NMR competition experiments were performed to determine if adenine, dATP, and suramin compete for the SAM binding site or influence the SAM binding with *Cp*-CobM. As a result, ligands competing by the same binding site, which influences saturation transfer from the protein, and differences in STD-AF are observed. In the competition experiments between SAM and dATP, or adenine and suramin, the signals from SAM are not observed in the difference spectrum. The adenine STD effect is reduced by approximately 19% (δ8.09 ppm: −18%; δ8.03 ppm: −20%) and the STD effect for dATP is reduced by approximately 11% (δ8.11 ppm: −11%). These results indicate significant competition between SAM, adenine, and dATP for the same binding site of *Cp*-CobM.

In contrast, competition with SAM reduced the suramin STD effect by only −0.06% (Supplementary Table S2), which is statistically insignificant when compared to the noise in the spectrum. This is probably because suramin does not interact with the SAM binding site of *Cp*-CobM. Suramin is a symmetric divalent polyanion with two naphthalene-trisulfonic acid head groups carrying the strong negatively charge that interacts with positively charged regions on protein surfaces and may cause changes in the protein secondary structure^23–25^.

### Determination of the dissociation constants between *Cp*-CobM and SAM, dATP, adenine, and suramin by fluorescence spectroscopy

Interaction between *Cp*-CobM with SAM, adenine, dATP and suramin was investigated using intrinsic tryptophan (Trp) fluorescence approach. The maximum emission of the intrinsic tryptophan fluorescence was centered at 303 nm. The Results of the *Cp*-CobM fluorescence quenching after ligand titration were used to determine the dissociation constant (K_d_), a nonlinear saturation curve approach and a modified Hill equation (Supplementary Fig. S7) was combined^26^.

The determined K_d_ values for all tested ligands showed the following order SAM > suramin > dATP > adenine demonstrated in Table 2.

**Table 2:** Dissociation constants of *Cp*-CobM with tested ligands and methyltransferases with SAM.

Protein	Organism	Ligand	K_d_ [µM]	Reference
CobM	*C*. *pseudotuberculosis*	SAM	1.4 ± 0.7	
		Adenine	17.8 ± 1.5	
		dATP	15.8 ± 2.0	
		Suramine	6.3 ± 1.1	
**Precorrin methyltransferases**				
CobA	*P*. *denitrificans*	SAM	6.3	^11^
CbiL/CobI	*M*. *Thermautotrophicus*	SAM	6.71 ± 0.8	^26^
**SAM-dependent methyltransferases**				
Adenine-N6 methyl- transferase	*B*. *subtilis*	SAM	1.2 ± 0.3	^27^
Adenine-specific DNA methyltransferase	*T*. *aquaticus*	SAM	2.0 ± 0.1	^28^
DNA-adenine-methylase	*E*. *coli*	SAM	6.5 ± 0.7	^29^
Aminoglycoside resistance methyl-transferase	*M*. *zionensis*	SAM	17.5	^30^
DNA adenine methyltransferase	*E*. *coli*	SAM	18.7 ± 1.81	^31^

The K_d_ value of the natural substrate (SAM) was 1.4 ± 0.7 µM, which demonstrated the greatest binding affinity when compared to the other precorrin methyltransferases, CobA and CbiL (6.3 and 6.71 µM)^11,27^ and lies within the range of known affinities for SAM dependent methyltransferases (1.24 to 18.7 µM)^28–32^.

Interestingly, the quenching behavior of the Trp residues in *Cp*-CobM shows differences between the tested ligands. Therefore, SAM, dATP, and adenine showed a similar quenching pattern. In contrast, suramin had a completely different effect on the Trp quenching; the difference in the quenching can be explained by the difference of the suramine size and the localization of the binding area. The STD NMR competition experiments showed that SAM, adenine, and dATP compete for the same binding area, the already known SAM binding site. These molecules are small and will not exert a strong influence on the Trp quenching. Suramin is a big molecule and can induce structural changes^33^ in the protein or, can serve as a shield to the Trp residues; both effects can induce the strong Trp quenching.

### Sequence analysis and homology modeling of *Cp*-CobM

A BLAST search for the *Cp*-CobM sequence against the atomic coordinates held in the PDB demonstrated the highest sequence identity of 51% with the *R*. *capsulatus*-CobM whose three-dimensional structure has been determined (PDB: 3NEI) and 42% identity with *B*. *megaterium*-CbiF (PDB: 1CBF), the RMSD between both structures is 1.079 (1157 atoms).

**Figure 4:**
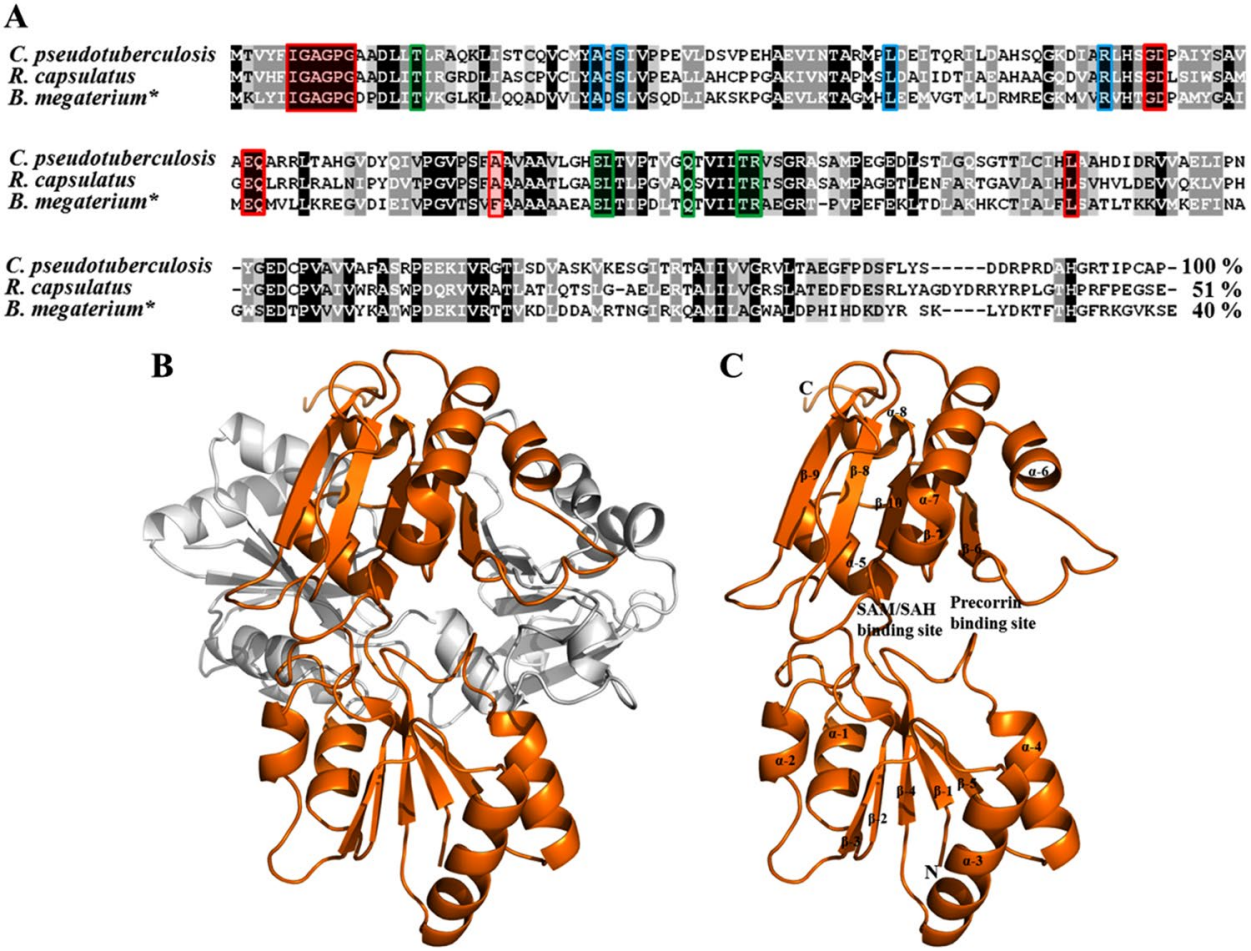
*Cp*-CobM homology model in ribbon representation and sequence comparison of bacterial CobM/CbiF proteins. (**A**) Sequence alignment of *Cp*-CobM, *R*. *capsulatus*-CobM and *B*. *megaterium*-CbiF (marked by asterisk). The red box indicates conserved residues involved in the SAM/SAH interaction. In green are indicated the residues involved in the dimer surface via hydrogen bonds. The blue indicates the residues involved in tetrapyrrole binding. (**B**) *Cp*-CobM dimer twisted 45° to each other, the monomer are coloured in orange and white. (**C**) *Cp*-CobM monomer, SAM/SAH and precorrin binding site highlighted.

Sequence alignments of *C*. *pseudotuberculosis*-CobM, *R*. *capsulatus*-CobM and *B*. *megaterium*-CbiF indicate the high conservation of regions important for function, such as the site of SAM/SAH interaction and the precorrin binding site, as well as residues in the dimer interface region (Fig. 4A).

A *Cp*-CobM homology model was generated based on the structure of the *R*. *capsulatus*-CobM, which was chosen based on the sequence identity of 51%. To obtain the protein models with the best quality for *Cp*-CobM, *Cp*-CobM-SAM and *Cp*-CobM SAH a cluster analysis was performed. For this analysis, the k-means method was used ranging from two to six. The correct cluster with the representative conformation was chosen by a combined analysis of the lowest Davies–Bouldin index (DBI) value and the highest pattern sequence based forecasting (pSF) (Supplementary Table S3). Additionally, the silhouette analysis (SI) was used to verify the best-formed cluster. To choose the representative conformation of the model, the distribution inside the cluster and the stability of the molecule over the MD simulation was used (supplementary Table S4). Calculation of the root mean square deviation (RMSD) and Radius of gyration (Rg) of Cα was determined to demonstrate the equilibrium and confluence of the systems (Supplementary Fig. S8).

The comparison of the *Cp*-CobM homology model with the *R*. *capsulatus-*CobM structure is presented (Supplementary Fig. S9). The *Cp*-CobM dimer is formed by two protein monomers that are twisted by 45° (Fig. 4B). The CobM monomer is composed of two α/β domains linked by a single coil forming a kidney shaped molecule^10^. The cavities formed between both domains are the SAM/SAH and the precorrin binding site. Both domains contain a five stranded β-sheet flanked by four α-helices^10^ (Fig. 4C).

### MD simulation of *Cp*-CobM complexed with SAM and SAH indicate dynamical flexibility during cofactor binding

**Figure 5:**
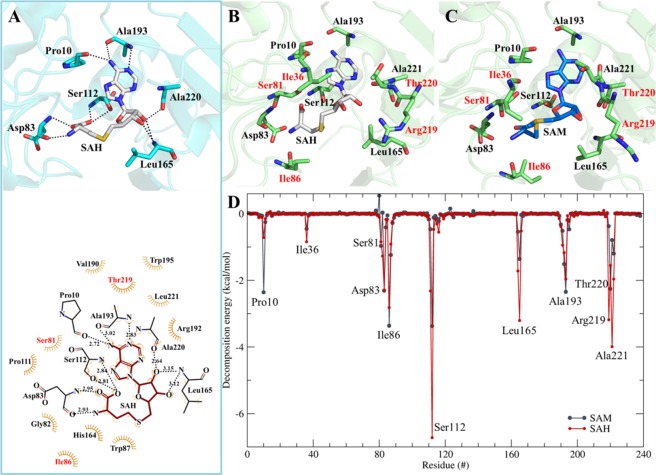
Comparison of the SAH and SAM binding sites in *C*. *pseudotuberculosis*-CobM and *R*. *capsulatus*-CobM. (**A**) (Upper panel) Interactions of SAH and *R*. *capsulatus*-CobM (PDB: 3NDC). (Lower panel) Ligplot representation indicating the hydrophobic interactions. (**B**) *Cp*-CobM homology model with SAH. (**C**) *Cp*-CobM homology model with SAM. Amino acids in red represent differences between the ligand binding in *Cp*-CobM and *R*. *capsulatus*-CobM. (**D**) Decomposition energy of *Cp*-CobM amino acids involved in the interaction with SAM (black) and SAH (red).

The SAM/SAH binding pocket of CobM proteins is located between the N- and C-terminal domains of the monomer. The residues involved in SAM/SAH binding are conserved between the species and include Pro10, Asp83, Ser112, Leu165, and Ala193 demonstrated for the *R*. *capsulatus*-CobM structure in complex with SAH. The protein ligand interaction is shown in a Ligplot figure (Fig. 5A) and demonstrates that the SAM interaction is stabilized by ten hydrogen bonds and eleven hydrophobic contacts. The adenine, ribose, and methionine parts of SAM are directly involved in the interaction (Fig. 5A). The NMR results demonstrated that adenine and dATP compete with SAM for the same binding site in the protein and there is a high possibility that they interfere mainly with the amino acids that interact with the SAM adenine part.

Docking experiments of SAH and SAM ligands in the *Cp*-CobM homology model and subsequently MD simulations indicated the interactions of amino acids involved in the SAH and SAM binding (Fig. 5B,C).

Comparison of the decomposition energies of both ligands indicates significant differences between SAM and SAH binding with *Cp*-CobM (Fig. 5D); these variations depend on the orientation of the ligand in the binding pocket and the number of hydrogen bonds formed. The largest differences can be observed for Pro10, Ser112, Leu165, Arg219 and Ala221. Depending on the ligands (SAH or SAM) for residues Ile36, Ser81, Asp83, Ile86, Leu165, Arg219, Thr220, and Ala221, a change in the coordination can be observed (Fig. 5B,C).

**Figure 6:**
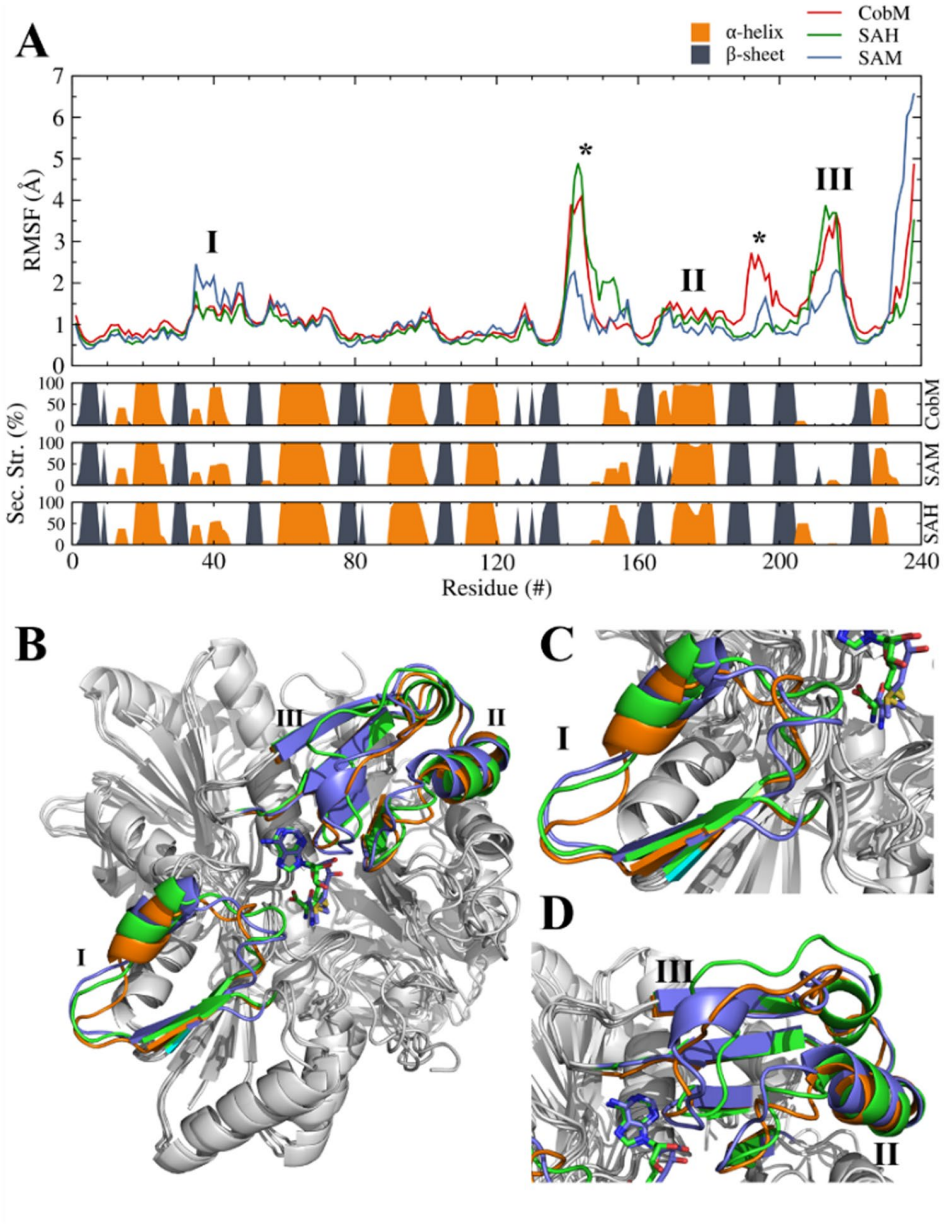
Root mean square fluctuation for each Cα atom of the *Cp*-CobM homology model calculated over the equilibrated trajectories for all the simulated systems (upper panel). (center panel) Averaged secondary structure elements of each simulation are presented for *Cp*-CobM and the complexes with SAH and SAM. (lower panel) The secondary structure is shown as a probability for each α-helix, β-sheet. Labelled regions are shown in the *Cp*-CobM homology model for comparison of the structural changes induced by ligand binding. *Cp*-CobM (orange), *Cp*-CobM-SAM (blue), *Cp*-CobM-SAH (green), Asterisks mark loop regions with greater fluctuations in the RMSF.

In the analog CbiF protein from *B*. *megaterium*, the transfer of the methyl group from SAM to precorrin-4 results in the release of SAH and precorrin-5, requiring significant conformational rearrangements in the enzyme^10^. To observe these conformational rearrangements in *Cp*-CobM model with and without ligands the Root Mean Square Fluctuation (RMSF) of the Cα atoms during the MD simulations were followed (Fig. 6).

The secondary structure and RMSF fluctuation of *Cp*-CobM and *Cp*-CobM-SAH are similar, but when compared with *Cp*-CobM-SAM complex they show significant differences in three regions that contain amino acids involved in the cofactor binding (Fig. 6B–D).

Since SAM binding initiates an opening movement of the active site pocket, the putative precorrin-4 binding region becomes more accessible. Changes in the secondary structure of CobM were also observed during the CD experiments. A comparison of the secondary structure content of *Cp*-CobM and *Cp*-CobM-SAM complex using CD spectroscopy and MD simulations showed a decreased amount of α-helices after binding of the SAM molecules and a slight increase in random coiled elements. The data for β-sheets are slightly different between CD spectroscopy and MD simulations (Supplementary Table S5).

The RMSF analysis identified two protein regions, which undergo significant fluctuations. These areas consist of loop regions and the movements are not considered to be induced by ligand binding (Fig. 6, peaks labeled by asterisks).

**Figure 7:**
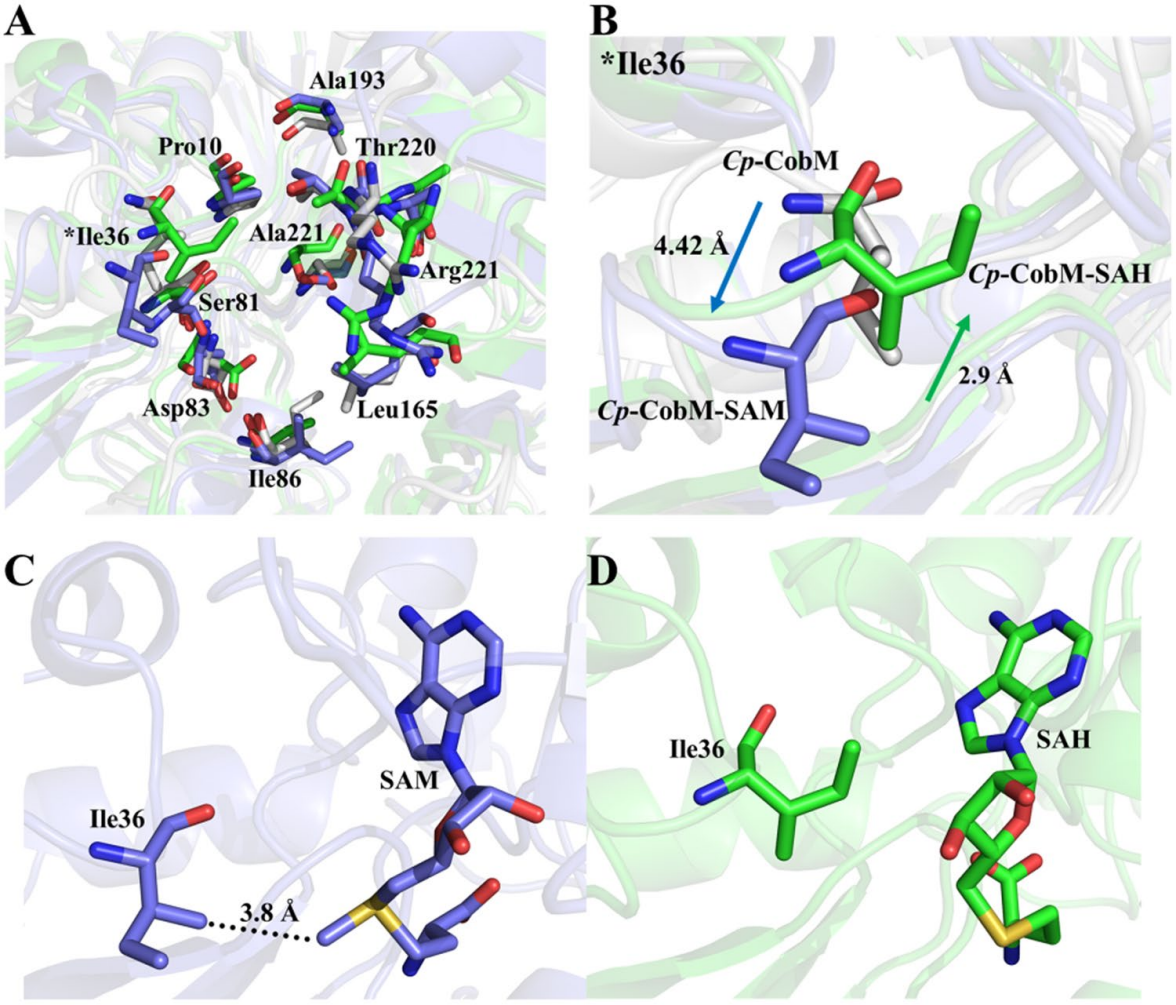
Comparison of the *Cp*-CobM SAM/SAH binding site. Highlighted are the amino acids involved in the cofactor binding. (**A**) *Cp*-CobM SAM/SAH binding site amino acids with and without ligand. (**B**) Ile36 movement during the cofactor binding. (**C**) Ile36 location and SAM. (**D**) Ile36 location and SAH.

The SAM/SAH binding area and the amino acids involved in the interaction to the cofactor undergo conformational changes during binding process. Figure 7A shows an overlay of the *Cp*-CobM amino acids involved in the binding with and without ligands and demonstrates the molecular movements after ligand interaction.

The strongest effect can be observed in Ile36 (Fig. 7A,B) located in the loop (Region I) with strong fluctuations during cofactor binding, which was described in Fig. 5. Interestingly, there is a displacement of approx. 4.42 Å after protein/cofactor binding (Fig. 7B colored by blue) related to the initial position (Fig. 7B side chain colored by white). During the SAH interaction, the same residue flips back 2.9 Å to the direction of the initial position (Fig. 7B colored by green). We suppose that Ile36 interacts with the CH_3_ group of SAM, which is transferred to precorrin-4. The distance between the amino acid and SAM CH_3_ group is 3.8 Å, which makes a hydrogen bond unlikely, but hydrophobic interaction possible. Figure 7C and D demonstrate the positions of Ile36 relating to SAM and SAH. The binding mode with SAM keeps the loop in a more open position, but after transferring the CH_3_ group the Ile36 shifts back to the initial position. These changes can be observed with a variation of the SAM/SAH binding pocket volume, *Cp*-CobM pocket has the biggest volume (401 Å^3^), followed by *Cp*-CobM-SAM (250 Å^3^) and *Cp*-CobM SAH (189 Å^3^) (Supplementary Fig. S10).

The supplementary video S1 shows the dynamics of the protein after binding to the cofactor.

## Conclusion

The complex biosynthesis of Vitamin B_12_ involves several enzymes and one of these enzymes, Precorrin-4 C(11)-methyltransferase (CobM), functions in the aerobic pathway. The sequence of *C*. *pseudotuberculosis* CobM shares 40–50% sequence identity with the structures of CobM from *R*. *capsulatus* and *B*. *megaterium*.

CobM exists physiologically as a dimer (Fig. 4B) and the two α/β domains of the monomer are linked by a short peptide (*Cp*-CobM Val107 to Ser112). The SAM/SAH binding site is located in a shallow cavity^10^; our results demonstrated upon binding of SAM that a relative hinge movement between the two domains occurs and this “opens” a cavity conformation, which is a prerequisite for precorrin-4 binding. A methyl group is extracted from SAM producing SAH followed by a further opening of the SAM/SAH binding site that allows the exit of SAH and precorrin-5.

MD simulations of a *Cp*-CobM homology model, CD, and NMR STD experiments all in the presence and in the absence of the ligands, identified regions in the protein that go through conformational rearrangements to facilitate the entrance of precorrin-4. CobM structure undergoes a conformational change that principally affects the access to the catalytic site increasing the accessibility.

SAM, dATP, adenine, and suramin interactions with *Cp*-CobM were also investigated using STD-NMR. Results of NMR competition experiments showed that adenine and dATP compete with SAM for the same binding site and, as revealed by CD spectroscopy, cause similar structural changes. Suramin affects SAM binding to CobM, however, since the competition effect with SAM is reduced, a different mechanism is likely involved.

The dissociation constants between *Cp*-CobM and the four ligands could be determined using fluorescence spectroscopy.

Understanding the conformational changes that this enzyme undergoes after cofactor and substrate binding sheds light on the molecular basis of the protein functionality giving perspective for drug development to combat caseous lymphadenitis.

## Material and Methods

### *In silico* analysis

The genome sequence of *C*. *pseudotuberculosis* strain 1002 (bv. ovis) and *C*. *pseudotuberculosis* strain CIP52.97 (bv. equi), both deposited in the NCBI GenBank database (http://www.ncbi.nlm.nih.gov/genbank/) were analyzed with the enzymes involved in the Vitamin B_12_ syntheses and Vitamin B_12_-dependent enzymes in perspective. Multiple CobM/CbiF sequences were retrieved from NCBI and sequence alignments were performed using MUSCLE^34^ and Box Shade (http://www.ch.embnet.org/software/BOX_form.htm) web servers. The protein sequence from *Cp*-CobM was retrieved from UniProt database (http://www.uniprot.org/) (D9QAC4).

### Cloning, expression and purification of *Cp*-CobM

The open reading frame of *Cp*-CobM was cloned into vector pD441-SR by DNA 2.0 (USA). The construct contained an N-terminal hexahistidine affinity tag and a TEV protease cleavage site (ENLYFQG). The kanamycin-resistant vector pD441-SR presents a T5 promoter inducible by IPTG and a high copy percentage provided by pUC origin of replication. CobM-pD441-SR (DNA 2.0) vectors were transformed into *E*. *coli* BL21 (DE3)T1 (Sigma-Aldrich, USA) chemical competent cells in LB medium, and grown at 37 °C for ~15 hours. Freshly prepared LB-medium + Kanamycin was inoculated with the preculture and grew at 37 °C up to OD_600_ of 0.6. The induction was performed with 1.0 mM IPTG and incubated at 37 °C with constant agitation for 4 hours. Following the cell culture was harvested using centrifugation under 4,000 rpm at 5 °C for 20 min, the supernatant was discarded and the *Cp*-CobM cell pellet was re-suspended in 20 mM Tris, pH 7.5, 500 mM NaCl, 10% (v/v) glycerol, 1 mM DTT.

The *Cp*-CobM containing cell-suspension was mixed with lysozyme and incubated on ice for 1 h. Afterwards, the cell-suspension was lysed by sonication in four sets of 30 s pulses of 30% amplitude with 10 s intervals. This method produced the crude cell extract, which was centrifuged under 8,000 rpm at 6 °C for 90 min. The supernatant containing *Cp*-CobM was loaded onto a Ni-NTA gravity flow column pre-equilibrated with 20 mM Tris, pH 7.5, 500 mM NaCl, 10% (v/v) glycerol, 1 mM DTT. The Ni-NTA column with bounded *Cp*-CobM was washed extensively with the same buffer containing 20, 40, and 80 mM imidazole. *Cp*-CobM was eluted with imidazole concentrations of 250 and 500 mM. The elution fractions containing *Cp*-CobM were pooled and applied onto a size exclusion column (Superdex 75 10/300 GL - GE Healthcare, USA), pre-equilibrated with buffer containing 20 mM K_2_HPO_4_/KH_2_PO_4,_ pH 7.5, 150 mM NaCl, 1 mM DTT. Protein purity was evaluated by 15% SDS-PAGE. The amount of recombinant *Cp*-CobM produced was around 20 mg/L.

Superdex 75 10/300 GL size exclusion column (GE Healthcare, USA) was calibrated using Albumin (MW: 65 kDa), Proteinase K (MW: 29.7 kDa) and Lysozyme (MW: 14.3 kDa). The column pre-equilibration was performed using a buffer solution containing: 20 mM K_2_HPO_4_/KH_2_PO_4,_ pH 7.5, 150 mM NaCl, 1 mM DTT, 1 mM EDTA, and 0.5 mM PMSF.

### Circular dichroism spectroscopy (CD)

For all CD measurements 15 repeated scans were performed with 5 of them used to establish the baseline. The wavelength range applied for far-UV spectra was from 200 nm to 260 nm in a time constant of 1 s and 100 nm/min continuous scanning mode, using a Jasco J-107 spectropolarimeter (Jasco, Japan). *Cp*-CobM was separately diluted in 5 mM K_2_HPO_4_/KH_2_PO_4_ to a concentration of 3.1 µM to investigate the influence of SAM, adenine, dATP, and suramin. The protein was incubated with a double molar excess (6.2 µM) for 2 h prior to the measurements. The results are presented in molar ellipticity [θ], according to:

$${[{\rm{\theta }}]}_{{\rm{l}}}={\rm{\theta }}/({\rm{c}}\,\ast \,{\rm{l}}\,\ast \,10\,\ast \,{\rm{n}})$$

where θ is the ellipticity measured at a given wavelength λ (deg), c is the protein concentration (mol L^−1^), l is the cell path length (cm) and, n is the number of amino acids. The results were analyzed and secondary structure amount determined using the CDpro software package^35^.

### Saturation transfer difference by nuclear magnetic resonance (STD-NMR)

Saturation Transfer Difference (STD) identifies binding events that occur in the rapid exchange of the saturation transfer^36^, i.e., interactions that possess a dissociation constant (K_d_) of µM to mM. Ligands in this range are of special relevance in the analysis of molecules that modulate protein function, rather than completely inhibiting it^37^ or, in preliminary screening of ligands for fragment-based drug design^38,39^.

NMR data was collected in a Bruker AVANCE III HD (Bruker, Germany), operating at 600 MHz for one hour and equipped with triple resonance cryoprobe with a pulsed field gradient. Bruker pulse sequence STDDIFFESGP.3 was used to perform the STD experiments with protein suppression by a spin lock filter. Each experiment was set with 64 scans, saturation time of two seconds (with four seconds of recycle delay) applied by a Gaussian pulse at 35 dBW with an acquisition time of 1.9 seconds in a slide window of 14 ppm. *Cp*-CobM saturation was set to 0.0 ppm, in order to keep a safety distance of 1000 Hz from any ligand signal, while off-resonance was set to 20 ppm. Protein concentration was 20 µM while the concentrations of the ligands SAM, dATP, adenine, and suramin were set to 400 µM. Samples were prepared in 20 mM K_2_HPO_4_/KH_2_PO_4_ pH 7.5, 150 mM NaCl with 10% D_2_O and transferred to 5 mm NMR tubes (Norell® Sample Vault Series™). STD effect is, calculated as:

$${\rm{STD}}=({{\rm{I}}}_{{\rm{off}}}-{{\rm{I}}}_{{\rm{on}}})/{{\rm{I}}}_{{\rm{off}}}$$

Where I_off_ and I_on_ are the integral of each ligand signal in the off- and on-resonance spectra, respectively. Data was collected in the STD experiment with each ligand individually and then for the competition between SAM and the other tested ligands.

### Fluorescence spectroscopy

Fluorescence spectroscopy were used to determine the K_d_ values for *Cp*-CobM with suramin, SAM, adenine, and dATP. A combination of a nonlinear saturation curve approach and a modified Hill equation were used to determine the K_d_ values, previously described in Coronado *et al*.^26^.

To measure the fluorescence of the intrinsic tryptophan we used a quartz cuvette with 3 mm path lengths (105.253-QS, Hellma, Mühlheim, Germany) and the experiments were performed on a QuantaMaster40 spectrofluorometer (PTI, Birmingham, USA). The background intensities were corrected for all spectra. The excitation and emission wavelength was chosen in 295 nm and in the range of 285–400 nm, respectively. The emission spectrum was recorded with increments of 1 nm and, each point in the emission spectrum represents the average of 10 accumulations. 10 μM final concentration of the protein was used in a volume of 1.5 ml in a buffer containing 20 mM K_2_HPO_4_/KH_2_PO_4,_ pH 7.5, 150 mM NaCl, 1 mM DTT. The interaction between suramin, SAM, adenine, and dATP with *Cp*-CobM was investigated. The titration was performed stepwise with a ligand stock solution (1.0 mM + 10 μM protein) and the measurement was performed after each titration. To fit a saturation binding curve^40^, the quenching of the *Cp*-CobM fluorescence, ΔF (F^max^ − F), at 303 nm for each titration point was used, based on equation (1)^41^:

1 $${\rm{Y}}={{\rm{B}}}_{{\rm{\max }}}[{\rm{Q}}]/{{\rm{K}}}_{{\rm{d}}}+[{\rm{Q}}]$$

where [Q] is the ligand concentration in solution, acting as a quencher, Y is the specific binding derived by measuring fluorescence intensity, B_max_ is the maximum amount of the complex *Cp*-CobM-ligand at saturation of the ligand and K_d_ is the equilibrium dissociation constant. The percentage of bound *Cp*-CobM, i.e. Y, derived from the fluorescence emission at the wavelength of maximum intensity, is plotted against the ligand concentrations.

Additionally, the data were fitted with a modified Hill equation, obtaining the following relation (2)^42,43^ where:

2 $$\mathrm{Log}({\rm{F}}-{{\rm{F}}}^{{\rm{\min }}})/({\rm{F}})={\rm{m}}\,\mathrm{log}\,{{\rm{K}}}_{{\rm{d}}}+{\rm{n}}\,\mathrm{log}[{\rm{Q}}]$$

F^min^ is the minimal fluorescence intensity in the presence of ligand; K_d_ is the equilibrium constant for the protein–ligand complex. The “binding constant” K is defined as the reciprocal of K_d_.

### Molecular dynamics and computational analysis

#### Initial structures

The atomic coordinates of *R*. *capsulatus*-CobM (PDB: 3NEI; sequence identity 51%) were used as a template for comparative modeling by the satisfaction of spatial restraints as implemented in the program Modeller 9v13^44^.

To obtain a high quality model of *Cp*-CobM, the protein structure was solvated in water and the system was neutralized with ion molecules. Subsequently, Molecular Dynamics (MD) simulations was performed (100 ns) and cluster analysis were used to generate a stable and representative *Cp*-CobM model. AutoDock Vina 1.1.12^45^ was used to perform docking studies for *Cp*-CobM-SAH and *Cp*-CobM-SAM. Polar hydrogens and partial charges were added to the protein using AutoDockTools program^46^, additionally, the rotational bonds in the ligands were defined.

The gridbox was used to define the search space near the ligand binding site. For each calculation, Autodock Vina scoring function ranked several structural poses. *Cp*-CobM-SAH and *Cp*-CobM-SAM complexes were used to initiate the MD simulations. Dimers were prepared by fitting the complexes models into the crystal structure, the biological form of the enzyme.

To prepare the ligands, Gaussian 09^47^ was used at the level of theory HF/6–31 G* to optimize and calculate their electrostatic potentials (ESP). Subsequently, the restrained electrostatic potential (RESP) charges were determined using antechamber program^48^ and the missing general amber force field (GAFF) parameters^49^ were obtained using parmchk program.

#### Parameterization for molecular dynamics simulations

The general setup for the *Cp-*CobM homology simulations in native form and with ligands were performed with some modification as described in Coronado *et al*.^26^.

MD simulations were performed using the AMBER16 program^50^, all atoms protein interaction was described using FF14SB force field^51^, while GAFF and RESP charges describe SAH and SAM ligands. The protonation state was settled up using the H++ web-server^52^, at pH 7.4. The experimental systems were neutralized with Na^+^ ions and settled in an octahedral or rectangular box of TIP3P water to at least 10 Å from any protein atom. Two steps of energy minimization for each system was performed to remove bad contacts from the initial structures. First, the energy minimization of the protein-complexes constrained (force constant of 50.0 kcal/mol.Å²) was achieved with 5,000 steepest descent followed by 5,000 conjugate gradient steps and by unconstrained energy minimization rounds (10,000 steps). After energy minimization, the system was gradually heated from 0 to 293 K for 300 ps under the constant number, volume, and temperature (NVT) ensemble, while the protein was restrained with a force constant of 25 kcal/mol.Å². Subsequently, an equilibration step was performed using the constant atom number, pressure, and temperature (NPT) ensemble for 2 ns. Finally, the production was run for 200 ns for each system and performed in NVT ensemble not having any restraints. The constant temperature (293 K) and pressure (1 atm) were controlled by Langevin coupling. The SHAKE constraints were applied to all bonds involving hydrogen atoms to allow a 2-fs dynamics time step. Long-range electrostatic interactions were calculated by the particle-mesh Ewald method (PME)^53^ using 8 Å cutoff.

#### Structural and dynamical analyses

The results were analyzed by the CPPTRAJ program^54^ of the AmberTools17 package. The system was visualized using VMD^55^ and Pymol^56^ programs. Root mean square deviation (RMSD) and Radius of gyration (Rg) of Cα were calculated to determine the system quality and stability (Supplementary Fig. S8).

Clustering analysis was performed with k-means method ranging from 2 to 6. To access the quality of clustering we used the DBI values and silhouette analyses. Protein flexibility was studied by Root Mean Square Fluctuation (RMSF) for the Cα atoms. The RMSF was calculated residue-by-residue over the equilibrated trajectories.

The generalized Born (GB)-Neck2^57^ implicit solvent model (igb = 8) was used to determine the interaction energy between protein and ligands.

The calculated Molecular Mechanics/Generalized Born Surface Area (MM/GBSA) energy showed the stability between the protein and the different ligands comprising the last 10 ns of the MD simulation, stripping all the solvent and ions.

The volume of the *Cp*-CobM SAM/SAM binding pocket was determined using the web tool POCASA1.1^58^.

## Supplementary information

<b>Binding studies of a putative <i>C. pseudotuberculosis</i> target protein from Vitamin B<sub>12</sub> Metabolism</b>

<b>Supplemental Video 1</b>

## Data Availability

The datasets generated during and/or analyzed during the current study are available from the corresponding author on reasonable request.

## References

[1] Raux E, Schubert HL, Warren MJ (2000). Biosynthesis of cobalamin (vitamin B12): a bacterial conundrum. Cell. Mol. Life Sci..

[2] Martens JH, Barg H, Warren M, Jahn D (2001). Microbial production of vitamin B12. Appl. Microbiol. Biotechnol..

[3] Klug G (2001). Beyond catalysis: vitamin B12 as a cofactor in gene regulation. Mol. Microbiol..

[4] Gopinath K, Moosa A, Mizrahi V, Warner DF (2013). Vitamin B12 metabolism in Mycobacterium tuberculosis. Future Microbiol..

[5] Banerjee R, Ragsdale SW (2003). The many faces of vitamin B12: catalysis by cobalamin-dependent enzymes. Annu. Rev. Biochem..

[6] Blanche F (1995). Vitamin B12: how the problem of its biosynthesis was solved. Angew. Chem. Int. Ed. Engl..

[7] Roth JR, Lawrence JG, Rubenfield M, Kieffer-Higgins S, Church GM (1993). Characterization of the cobalamin (vitamin B12) biosynthetic genes of Salmonella typhimurium. J. Bacteriol..

[8] Raux E, Schubert HL, Roper JM, Wilson KS, Warren MJ (1999). Vitamin B12: insights into biosynthesis’s Mount Improbable. Bioorg. Chem..

[9] Warren MJ, Raux E, Schubert HL, Escalante-Semerena JC (2002). The biosynthesis of adenosylcobalamin (vitamin B12). Nat. Prod. Rep..

[10] Schubert HL, Wilson KS, Raux E, Woodcock SC, Warren MJ (1998). The X-ray structure of a cobalamin biosynthetic enzyme, cobalt-precorrin-4 methyltransferase. Nat. Struct. Mol. Biol..

[11] Blanche F, Debussche L, Thibaut D, Crouzet J, Cameron B (1998). Purification and characterization of S-adenosyl-L-methionine: uroporphyrinogen III methyltransferase from Pseudomonas denitrificans. J. Bacteriol..

[12] Warren MJ, Roessner CA, Santander PJ, Scott AI (1990). The *Escherichia coli* cysG gene encodes S-adenosylmethionine-dependent uroporphyrinogen III methylase. Biochem. J..

[13] Roessner CA, Williams HJ, Scott AI (2005). Genetically engineered production of 1-desmethylcobyrinic acid, 1-desmethylcobyrinic acid a, c-diamide, and cobyrinic acid a, c-diamide in *Escherichia coli* implies a role for CbiD in C-1 methylation in the anaerobic pathway to cobalamin. J. Biol. Chem..

[14] Ayers JL (1997). Caseous lymphadenitis in goats and sheep: a review of diagnosis, pathogenesis, and immunity. J. Am. Vet. Med. Assoc..

[15] Dorella FA, Pacheco LG, Oliveira SC, Miyoshi A, Azevedo V (2006). Corynebacterium pseudotuberculosis: microbiology, biochemical properties, pathogenesis and molecular studies of virulence. Vet. Res..

[16] Silva A (2011). Complete genome sequence of Corynebacterium pseudotuberculosis I19, a strain isolated from a cow in Israel with bovine mastitis. J. Bacteriol..

[17] Ruiz JC (2011). Evidence for reductive genome evolution and lateral acquisition of virulence functions in two Corynebacterium pseudotuberculosis strains. PloS one..

[18] Costa LR, Spier SJ, Hirsh DC (1998). Comparative molecular characterization of Corynebacterium pseudotuberculosis of different origin. Vet. Microbiol..

[19] Sutherland. SS, Hart RA, Buller NB (1996). Genetic differences between nitrate-negative and nitrate-positive *C. pseudotuberculosis* strains using restriction fragment length polymorphisms. Vet. Microbiol..

[20] Savvi S (2008). Functional characterization of a vitamin B12-dependent methylmalonyl pathway in *Mycobacterium tuberculosis*: implications for propionate metabolism during growth on fatty acids. J. Bacteriol..

[21] Koutmos M, Datta S, Pattridge KA, Smith JL, Matthews RG (2009). Insights into the reactivation of cobalamin-dependent methionine synthase. Proc. Natl. Acad. Sci. USA.

[22] Pejchal R, Ludwig ML (2005). Cobalamin-independent methionine synthase (MetE): a face-to-face double barrel that evolved by gene duplication. PLoS Biol..

[23] Monteiro RQ, Campana PT, Melo PA, Bianconi ML (2004). Suramin interaction with human α-thrombin: inhibitory effects and binding studies. Int. J. Biochem. Cell. Bio..

[24] Fini C (1996). Boar sperm proacrosin infrared investigation: Secondary structure analysis after autoactivation and suramin binding. Biochem. Mol. Med..

[25] Fleck SL (2003). Suramin and suramin analogues inhibit merozoite surface protein-1 secondary processing and erythrocyte invasion by the malaria parasite Plasmodium falciparum. J. Biol. Chem..

[26] Coronado MA (2018). Zika virus NS2B/NS3 proteinase: A new target for an old drug-Suramin a lead compound for NS2B/NS3 proteinase inhibition. Antiviral Res..

[27] Frank S (2007). Elucidation of Substrate Specificity in the Cobalamin (Vitamin B12) Biosynthetic Methyltransferases, structure and function of the C20 methyltransferase (CbiL) from *Methanothermobacter thermautotrophicus*. J. Biol. Chem..

[28] Maravić G (2003). Mutational analysis defines the roles of conserved amino acid residues in the predicted catalytic pocket of the rRNA: m6A methyltransferase ErmC′. J. Mol. Biol..

[29] Schluckebier G (1997). Differential binding of S-adenosylmethionine S-adenosylhomocysteine and Sinefungin to the adenine-specific DNA methyltransferase M. TaqI1. J. Mol. Biol..

[30] Bergerat A, Guschlbauer W (1990). The double role of methyl donor and allosteric effector of S-adenosyl-methionine for Dam methylase of *E. coli*. Nucleic Acids Res..

[31] Savic M (2008). Critical residues for cofactor binding and catalytic activity in the aminoglycoside resistance methyltransferase Sgm. J. Bacteriol..

[32] Mashhoon N (2004). Functional characterization of *Escherichia coli* DNA adenine methyltransferase, a novel target for antibiotics. J. Biol. Chem..

[33] Middaugh CR (1992). Nature of the interaction of growth factors with suramin. Biochemistry..

[34] Edgar RC (2004). MUSCLE: a multiple sequence alignment method with reduced time and space complexity. BMC bioinformatics.

[35] Sreerama N, Woody RW (2000). Estimation of protein secondary structure from circular dichroism spectra: comparison of CONTIN, SELCON, and CDSSTR methods with an expanded reference set. Anal. Biochem..

[36] Mayer M, Meyer B (1999). Characterization of ligand binding by saturation transfer difference NMR spectroscopy. Angewandte Chemie-Int..

[37] Qin J, Gronenborn AM (2014). Weak protein complexes: challenging to study but essential for life. FEBS J..

[38] Murray CW, Blundell TL (2010). Structural biology in fragment-based drug design. Curr. Opin. Struct. Biol..

[39] Harner MJ, Frank AO, Fesik SW (2013). Fragment-based drug discovery using NMR spectroscopy. J. Biomol. NMR.

[40] Johnson ML, Frasier SG (1985). Nonlinear least-squares analysis. Meth Enzymol.

[41] Shaikh SMT, Seetharamappa J, Ashoka S, Kandagal PB (2007). A study of the interaction between bromopyrogallol red and bovine serum albumin by spectroscopic methods. Dyes Pigm.

[42] Wang G, Liu X, Yan C, Bai G, Lu Y (2015). Probing the binding of trypsin to glutathione-stabilized gold nanoparticles in aqueous solution. Colloids Surf B Biointerfaces.

[43] Ahumada M (2017). Association models for binding of molecules to nanostructures. Analyst.

[44] Eswar N (2007). Comparative Protein Structure Modeling With MODELLER. Curr. Protoc. Bioinformatics.

[45] Trott O, Olson AJ (2009). AutoDock Vina: Improving the speed and accuracy of docking with a new scoring function, efficient optimization, and multithreading. J. Comput. Chem..

[46] Morris GM (2009). AutoDock4 and AutoDockTools4: Automated docking with selective receptor flexibility. J. Comput. Chem..

[47] Frisch, D. J. *et al*. Gaussian 09, Revision C.01, http://gaussian.com/ (2009).

[48] Wang J, Wang W, Kollman PA, Case DA (2006). Automatic atom type and bond type perception in molecular mechanical calculations. J. Mol. Graph. Model.

[49] Wang JM, Wolf RM, Caldwell JW, Kollman PA, Case DA (2004). Development and testing of a general amber force field. J. Comput. Chem..

[50] Case DA (2005). The Amber biomolecular simulation programs. J. Computat. Chem..

[51] Maier JA (2015). ff14SB: Improving the Accuracy of Protein Side Chain and Backbone Parameters from ff99SB. J. Chem. Theory Comput..

[52] Gordon JC (2005). H++: a server for estimating pKas and adding missing hydrogens to macromolecules. Nucleic Acids Res..

[53] Darden T, York D, Pedersen L (1993). Particle mesh Ewald: An N⋅ log (N) method for Ewald sums in large systems. J. Chem. Phys..

[54] Roe DR, Cheatham TE (2013). PTRAJ and CPPTRAJ: software for processing and analysis of molecular synamics trajectory data. J. Chem. Theory Com..

[55] Humphrey W, Dalke A, Schulten K (1996). VMD: Visual molecular dynamics. J. Mol. Graph..

[56] DeLano WL (2002). Pymol: An open-source molecular graphics tool. CCP4 Newsletter On Protein Crystallography.

[57] Nguyen H, Roe DR, Simmerling C (2013). Improved Generalized Born Solvent Model Parameters for Protein Simulations. J. Chem. Theory Comput..

[58] Yu J, Zhou Y, Tanaka I, Yao M (2010). Roll: A new algorithm for the detection of protein pockets and cavities with a rolling probe sphere. Bioinformatics..

