# Supplementary material for: Binding studies of a putative C. pseudotuberculosis target protein from Vitamin B12 Metabolism
Source: Sci Rep. 2019 Apr 23;9:6350. doi: 10.1038/s41598-019-42935-y (PMC6478909; doi:10.1038/s41598-019-42935-y)
Supplement: Supplementary file 1 — Binding studies of a putative C. pseudotuberculosis target protein from Vitamin B12 Metabolism [file 41598_2019_42935_MOESM1_ESM.docx]

**Supplementary Information**

**Binding studies of a putative *C. pseudotuberculosis* target protein from Vitamin B_12_ Metabolism**

Rafaela dos S. Peinado^a+^, Danilo S. Olivier^a+^, Raphael J. Eberle^a+^, Fabio R. de Moraes^a^, Marcos S. Amaral^b^, Raghuvir K. Arni^a*^, Monika A. Coronado^a*^

^a^Multiuser Center for Biomolecular Innovation, Departament of Physics, Instituto de Biociências
Letras e Ciências Exatas (Ibilce), Universidade Estadual Paulista (UNESP), São Jose do Rio Preto-SP, 15054-000, Brazil.

^b^Institute of Physics, Federal University of Mato Grosso do Sul, Campo Grande-MS, 79090-700, Brazil.

#Authors contributed equally to the work

*Correspondence to [monikacoronado@gmail.com](mailto:monikacoronado@gmail.com) or [raghuvir.arni@unesp.br](mailto:raghuvir.arni@unesp.br)

Table of contents

Supplemental Figure 1. Protein structures of transmethylases involved in Vitamin B_12_ biosynthesis.

Supplemental Figure 2. Sequence alignment of the six methyltransferases involved in *C. pseudotuberculosis* Vitamin B_12_ synthesis.

Supplemental Figure 3. Arrangement of cobalamin biosynthetic genes in bacteria species.

Supplemental Figure 4. Expression profile and purification of *Cp*-CobM.

Supplemental Figure 5. SEC calibration and comparison with *Cp*-CobM chromatogram.

Supplemental Figure 6. Comparison of *Cp*-CobM homology model and *B. megaterium*-Cbif active site.

Supplemental Figure 7. Fluorescence spectroscopy of Trp at 295 nm in *Cp*-CobM under the influence SAM, adenine, dATP and suramin.

Supplemental Figure 8. Time dependent modifications of *Cp*-CobM homology model in the absence and in the presence of SAM and SAH.

Supplemental Figure 9. Superposition of the *Cp*-CobM homology model in and the *R. capsulatus* CobM structure.

Supplemental Figure 10. Comparison of *Cp*-CobM SAM/SAH binding pocket volume.

Supplemental Table 1. Enzymes to be involved in vitamin B_12_ biosynthesis in *C. pseudotuberculosis* strain 1002 (bv. ovis).

Supplemental Table 2. Average STD effect for suramin in NMR competition experiments of *Cp*-CobM with SAM.

Supplemental Table 3. Cluster analysis of *Cp*-CobM, *Cp*-CobM-SAM and *Cp*-CobM-SAH models

Supplemental Table 4. Conformations in the chosen cluster and the percentage of division.

Supplemental Table 5. Comparison of the secondary structure content of *Cp*-CobM determined by MD simulations and CD spectroscopy.

Supplemental Video 1. *Cp*-CobM molecular movements after SAM binding.

**
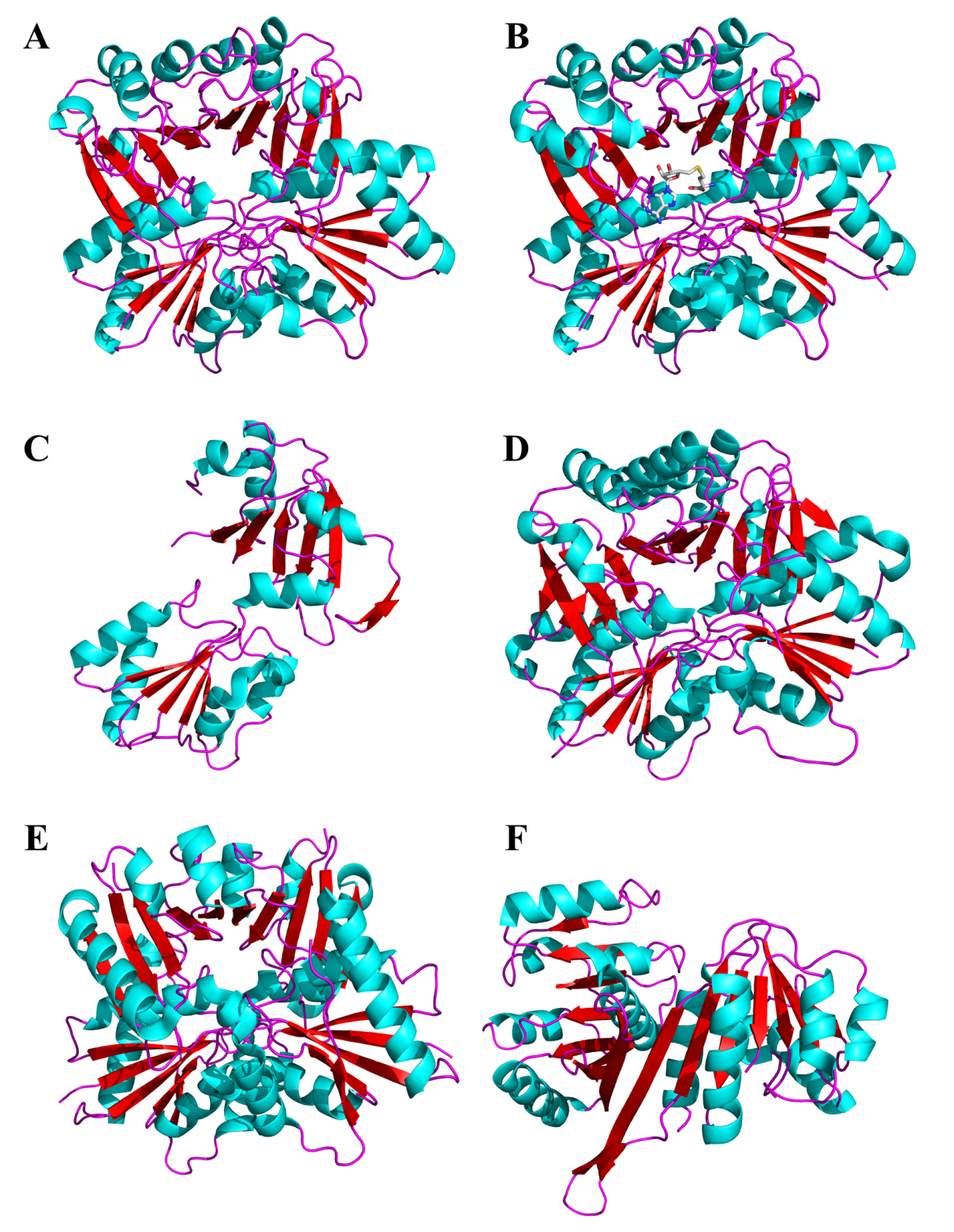
**

**Figure S1.** Protein crystal structures of transmethylases involved in Vitamin B_12_ biosynthesis, deposited in the PDB and *Cp*-CobM homology model. The protein structures are shown in ribbon representation; α-helices are colored in turquoise, β-sheets in red and loops in pink. **A:** Homology model of *Cp*-MutY. **B:** Crystal structure of *R. capsulatus* CobM in complex with SAH (PDB: 3NDC). **C:** Crystal structure of *P. aeruginosa* CobA (PDB: 2YBQ). **D:** Crystal structure of *R. capsulatus* CobJ (PDB: 3NUT). **E:** Crystal structure of *R. capsulatus* CobF (PDB: 3ND1). **F:** Crystal structure of *C. diphtheria* CobL (PDB: 3HM2).


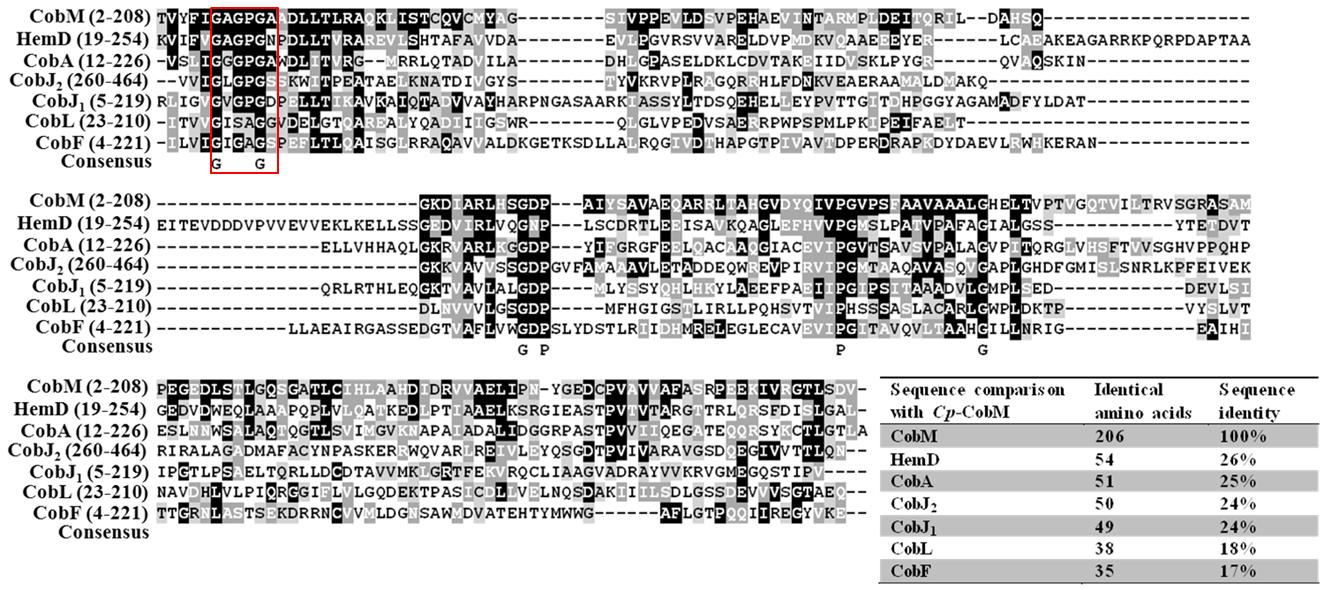


**Figure S2.** Sequence alignment of the six methyltransferases involved in *C. pseudotuberculosis* Vitamin B_12_ synthesis. The methyltransferase domains are considered for the sequence alignment. CobJ contain two methyl transferase domains, CobJ_1_ and CobJ_2_. The red box indicate the glycine-rich sequence GAGPGD motif. Black shading lable conserved residues, dark grey shading highly related residues and light grey shading related residues. The table indicate the sequence identity of each methyltransferase to the *Cp*-CobM sequence.

**
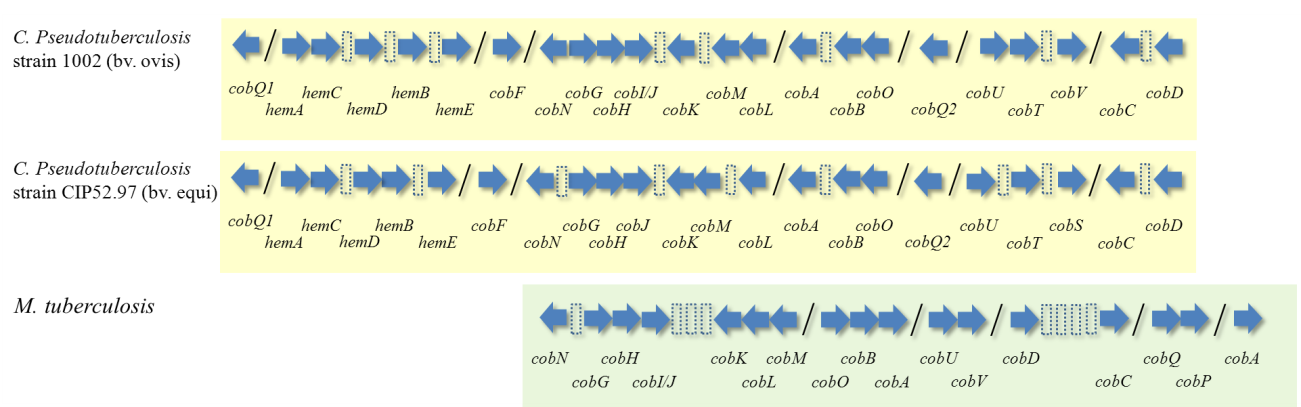
**

**Figure S3.** Arrangement of cobalamin biosynthetic genes in *C. pseudotuberculosis* strain 1002 (bv. ovis), C. pseudotuberculosis strain CIP52.97 (bv. equi) and *M. tuberculosis* genome.

**
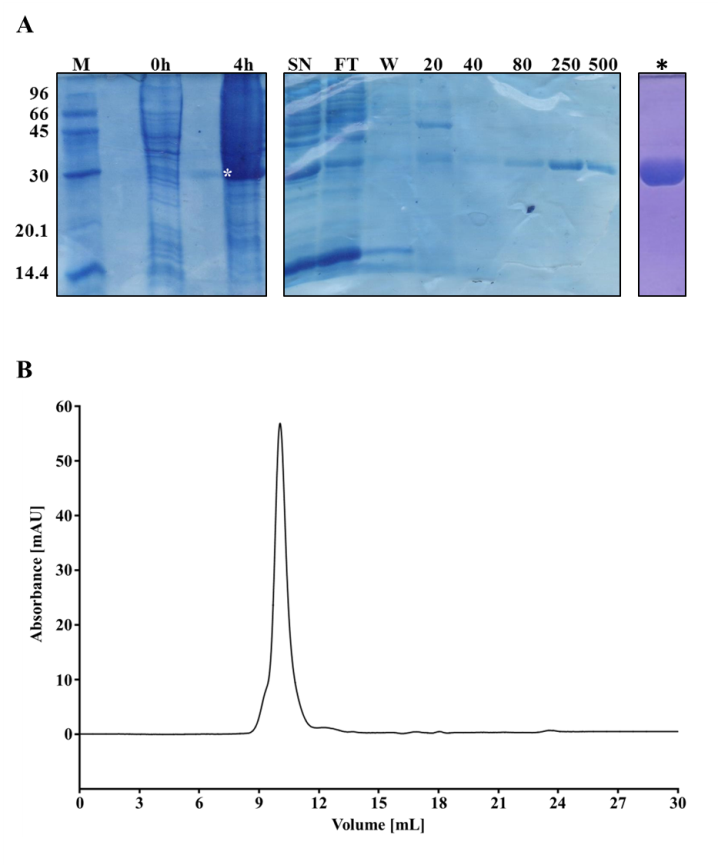
**

**Figure S4.** Expression profile and purification of *Cp*-CobM. **A:** SDS-PAGE of the expression profile of *Cp*-CobM in *E. coli* BL21 T1 cells. Expressed at 37° C for 4h, induced with 1.0 mM IPTG respectively. 0h: induction, 4h: after 4h expression the white asterisk lable the *Cp*-CobM protein band. SDS-PAGE analysis of *Cp*-CobM after Ni-NTA column. SN: supernatant after cell disruption, FT: flow through, W: washing step without imidazole, numbers indicate imidazole concentration in mM. SDS-PAGE of *Cp*-CobM after size exclusion chromatography, which indicate the purity of the protein. **B:** Chromatogram of size exclusion chromatography of *Cp*-CobM, elute at around 10 ml (*), which corresponds a dimer of *Cp*-CobM.


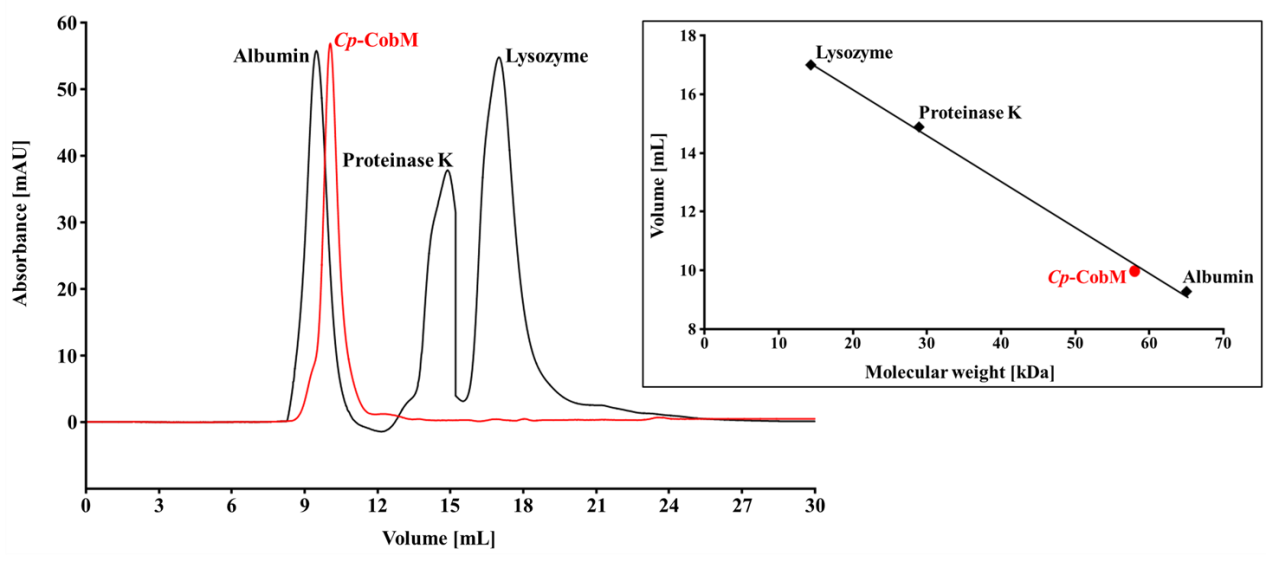


**Figure S5.** Calibration of the SEC column used for the *Cp*-CobM purification. The chromatogram show the elution peaks of Albumin (65 kDa), Proteinase K (29.7 kDa) and Lysozyme (14.3 kDa). *Cp*-CobM elute at around 10 ml, the protein monomer has a MW of around 29 kDa, but the protein elute cleary as dimer near a MW of 60 kDa as demonstrated in the linear elution plot.


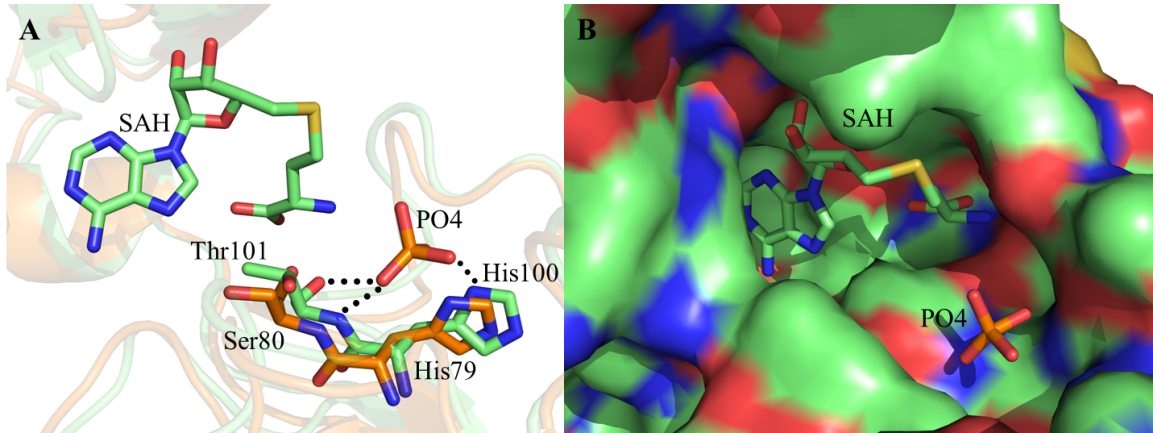


**Figure S6.** Comparison of *Cp*-CobM homology model and *B. megaterium*-Cbif protein structure (PDB: 1CBF) active site with bound phosphate ion. **A:** Amino acids involved in phosphate ion binding represented in sticks. **B:** Surface view of *B. megaterium*-Cbif with SAH and phosphate ion.


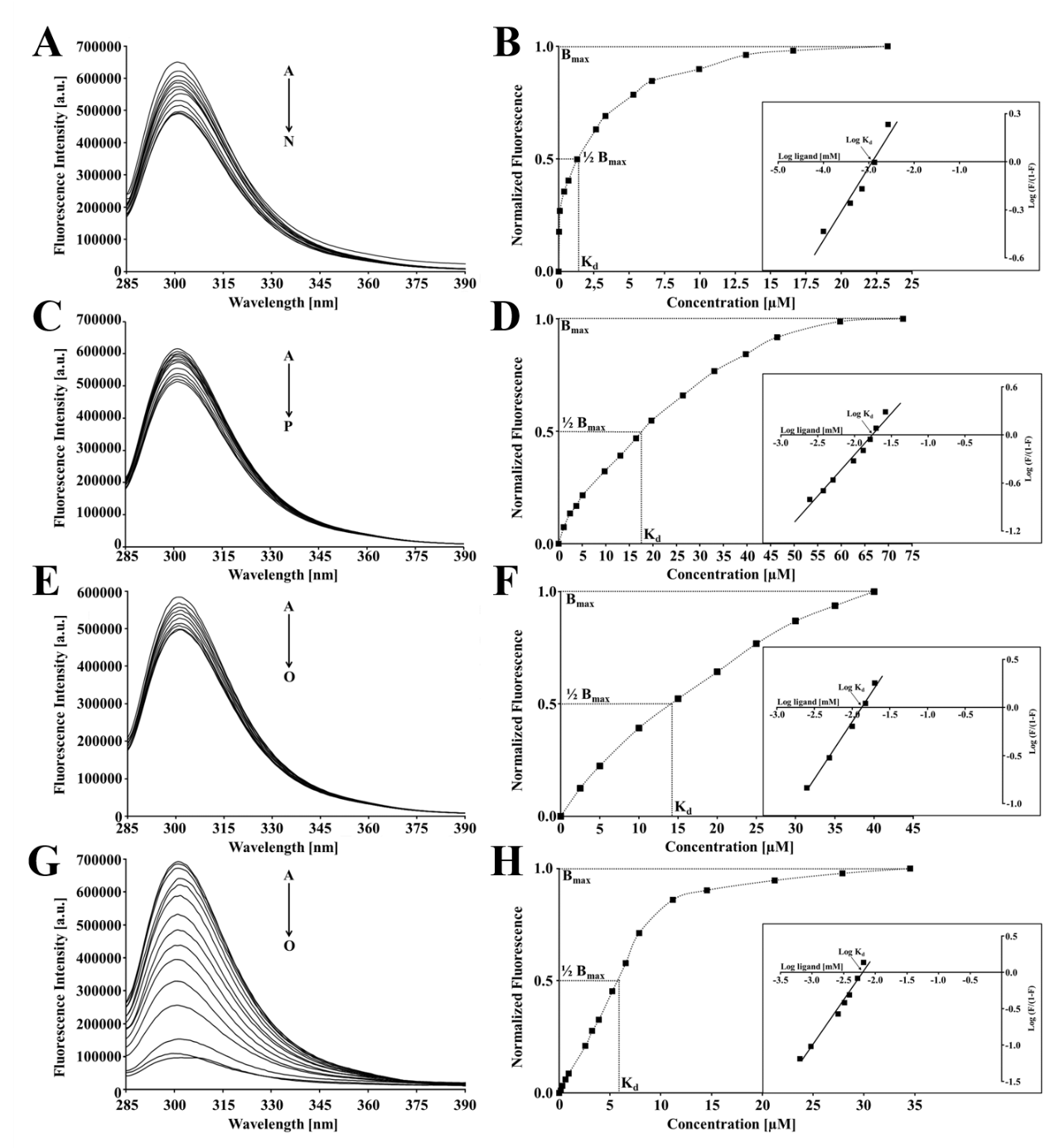


**Figure S7.** Fluorescence spectroscopy of Trp at 295 nm in *Cp*-CobM under the influence SAM, adenine, dATP and suramin. **A:** Titration of SAM **B:** Binding saturation curve and modified Hill equation determined a K_d_ value of 1.4 ± 0.7 µM for SAM and *Cp*-CobM. **C:** Titration of adenine from **D:** Binding saturation curve and modified Hill equation determined a K_d_ value of 17.8 ± 1.5 µM for adenine and *Cp*-CobM. **E:** Titration of dATP. **F:** Binding saturation curve and modified Hill equation determined a K_d_ value of 15.8 ± 2.0 µM for dATP and *Cp*-CobM. **G:** Titration of Suramin. **H:** Binding saturation curve and modified Hill equation determined a K_d_ value of 6.3 ± 1.1 µM for suramin and *Cp*-CobM.


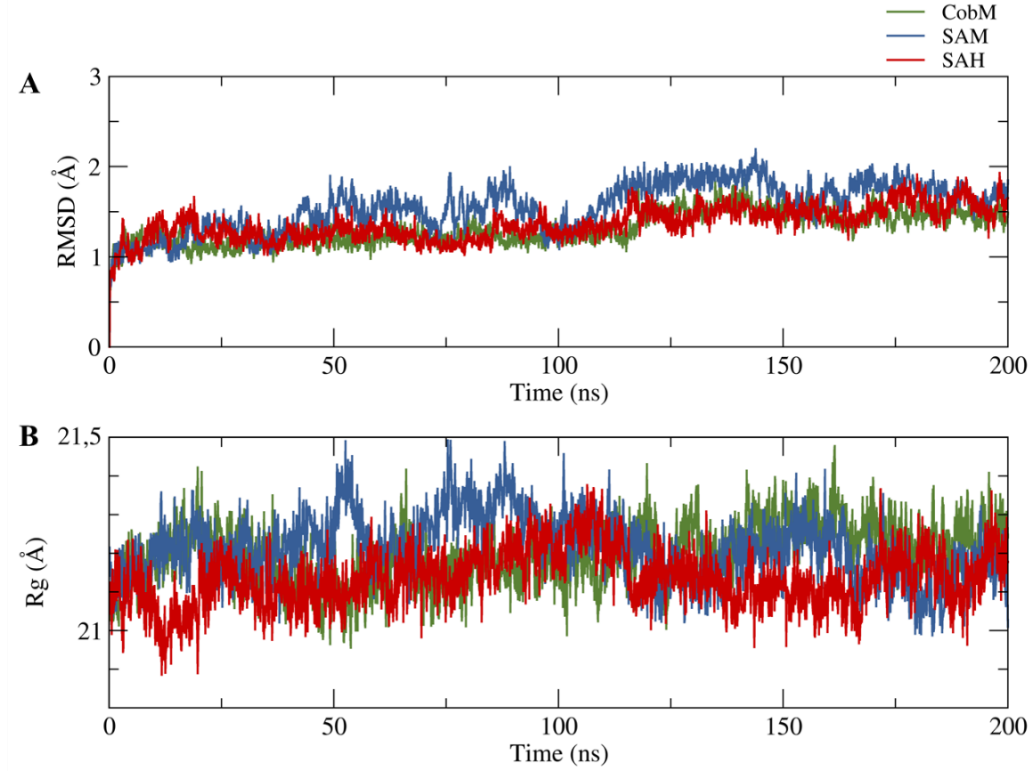


**Figure S8.** Time dependent modifications of *Cp*-CobM homology model in the absence and in the presence of SAM and SAH. **A:** RMSD as function of time. **B:** Rg as function of time.


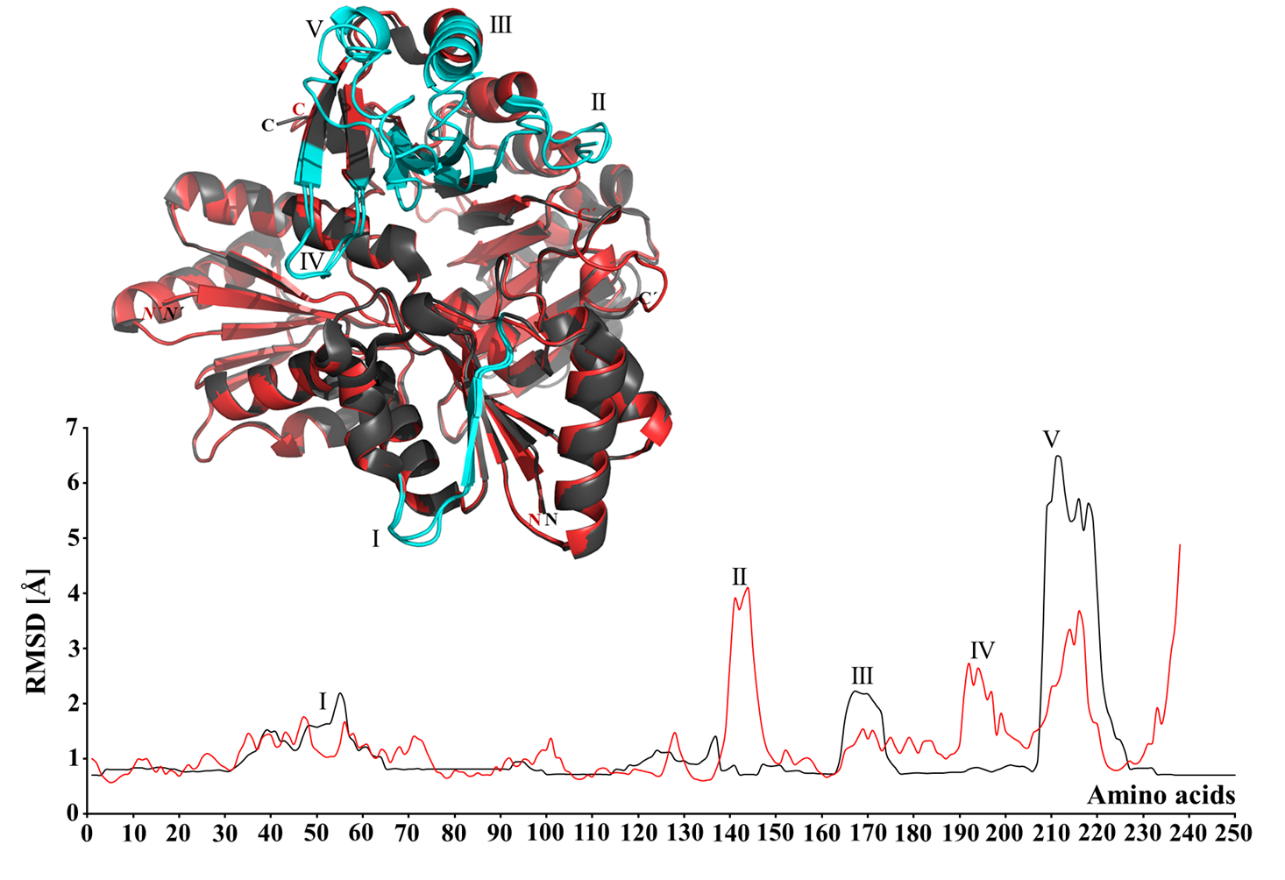


**Figure S9.** Homology model of *C. pseudotuberculosis*-CobM. Superposition of the *Cp*-CobM model in red and the *R. capsulatus*-CobM structure (PDB: 3NEI) in dark grey. The rmsd per aminoacid is shown and the regions with strong differences are marked I-V in the rmsd plot and structural overlay (light blue).

**
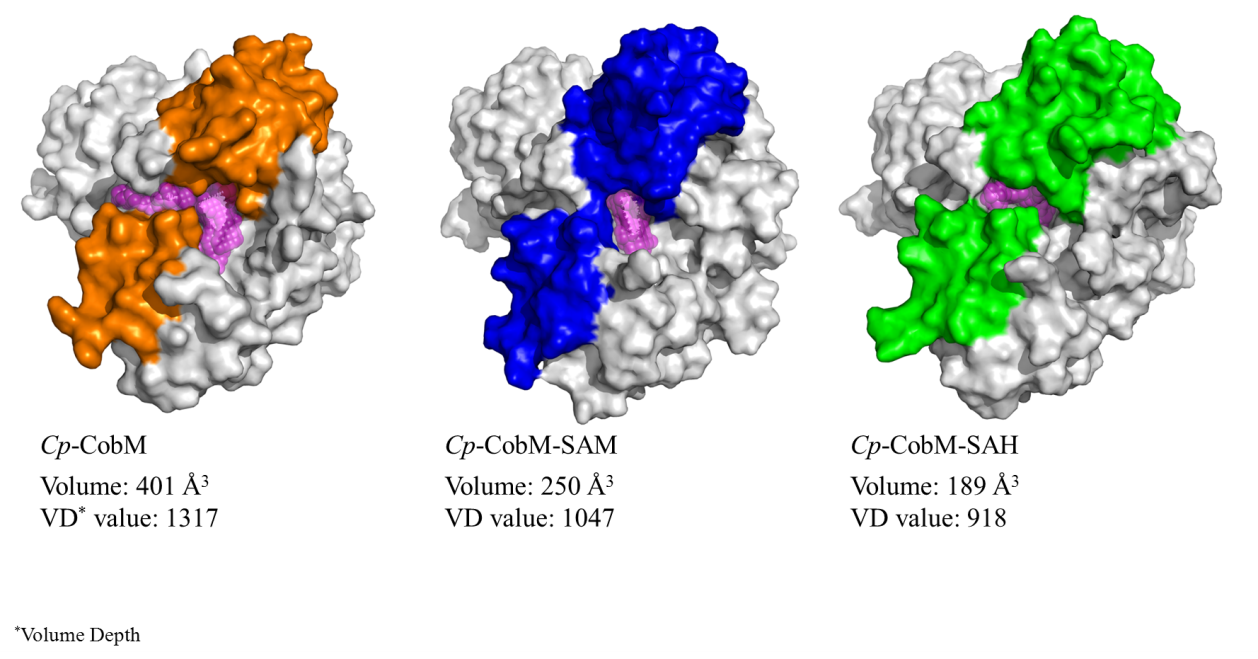
**

**Figure S10.** Comparison of *Cp*-CobM SAM/SAH binding pocket volume. Cp-CobM, Cp-CobM-SAM and Cp-CobM-SAH were investigated. The protein regions, which undergo conformational changes are colored and the pocket volume is represented as a magenta sphere.

**Table S1.** Enzymes to be involved in vitamin B12 biosynthesis in *C. pseudotuberculosis* strain 1002 (bv. ovis)

|  | Gene name | Putative function | *C. pseudotuberculosis* |
| --- | --- | --- | --- |
| ADL09770 | *hemA* | Glutamyl-tRNA reductase |  |
| ADL09773 | *hemB* | Delta-aminolevulinic acid dehydratase |  |
| ADL09771 | *hemC* | Porphobilinogen deaminase |  |
| ADL09772 | *hemD* | Uroporphyrinogen-III synthase |  |
| ADL09776 | *hemE* | Uroporphyrinogen decarboxylase |  |
| ADL10112 | *cobF* | Precorrin-6A synthase |  |
| ADL10495 | *cobN* | Cobaltochelatase |  |
| ADL10496 | *cobG* | Precorrin-3B synthase |  |
| ADL10497 | *cobH* | Precorrin-8X methyl mutase |  |
| ADL10498 | *cobI/J* | Precorrin-3B methylase / Precorrin-3B C(17)-methyltransferase |  |
| ADL10499 | *cobK* | Precorrin-6A reductase |  |
| ADL10500 | *cobM* | Precorrin-4 C11-methyltransferase |  |
| ADL10501 | *cobL* | Precorrin-6Y C5,15-methyltransferase |  |
| ADL10757 | *cobA/CysG* | Uroporphyrinogen-III methylase |  |
| ADL10761 | *cobB* | Hydrogenobyrinate a,c-diamide synthase |  |
| ADL10762 | *cobO* | Cob (I) yrinic acid a,c-diamide adenosyltransferase |  |
| ADL10899 | *cobU/cobP* | Bifunctional adenosylcobalamin biosynthesis protein |  |
| ADL10900 | *cobT* | Nicotinate-nucleotide--dimethylbenzimidazole phosphoribosyltransferase |  |
| ADL10901 | *cobS/cobV* | Adenosylcobinamide-GDP ribazoletransferase |  |
| ADL10954 | *cobC/* *CpC231_1488* | Bifunctional RNase H/acid phosphatase |  |
| ADL10961 | *cobD* | Cobalamin biosynthesis protein CobD |  |
| APQ55354 | *cobQ1* | Cobyric acid synthase |  |
| APQ56476 | *cobQ2* | Cobyric acid synthase |  |
| - | *cobE* | - | - |

**Table S2.** Average STD effect for suramin in NMR competition experiments with SAM

| Suramin | | | | | | | | |
| --- | --- | --- | --- | --- | --- | --- | --- | --- |
| Average  change | STD @ δ9.3288 ppm | STD @ δ8.5844 ppm | STD @ δ8.2203  ppm | STD @ δ7.8084 ppm | STD @ δ7.6534 ppm | STD @ δ7.4498 ppm | STD @ δ7.2414 ppm | STD @ δ2.0935 ppm |
| -0.06% | -0.33% | -0.44% | -0.04% | -0.22% | 0.08% | -0.05% | -0.07% | 0.09% |

**Table S3.** Cluster analysis of *Cp*-CobM, *Cp*-CobM-SAM and *Cp*-CobM-SAH models, the chosen clusters labeled in yellow.

| **Cluster** | **DBI** | **pSF** | **SSR/SST** | **<Si>** |
| --- | --- | --- | --- | --- |
| *Cp*-CobM | | | | |
| 2 | 1.32 | 5420.92 | 0.35 | 0.316 |
| 3 | 1.52 | 4468.43 | 0.47 | 0.266 |
| 4 | 1.79 | 3501.69 | 0.51 | 0.205 |
| 5 | 1.85 | 2949.94 | 0.54 | 0.192 |
| 6 | 1.97 | 2610.55 | 0.57 | 0.185 |
| *Cp*-CobM-SAM | | | | |
| 2 | 1.51 | 2687.11 | 0.21 | 0.267 |
| 3 | 1.97 | 2256.98 | 0.31 | 0.207 |
| 4 | 1.55 | 2797.30 | 0.46 | 0.247 |
| 5 | 1.52 | 2420.94 | 0.49 | 0.255 |
| 6 | 1.58 | 2227.24 | 0.53 | 0.236 |
| *Cp*-CobM-SAM | | | | |
| 2 | 1.37 | 5105.48 | 0.34 | 0.307 |
| 3 | 1.64 | 3490.12 | 0.41 | 0.235 |
| 4 | 1.67 | 2746.59 | 0.45 | 0.221 |
| 5 | 2.06 | 2436.51 | 0.49 | 0.172 |
| 6 | 1.82 | 2209.08 | 0.53 | 0.194 |

**Table S4.** Conformations in the chosen cluster and the percentage of division.

| System | Conformation | Frame | Percentage (%) |
| --- | --- | --- | --- |
| Cobm | *C_0_ | 868 | 57.7 |
|  | C_1_ | 8159 | 42.3 |
| Cobm-SAM | *C_0_ | 6248 | 79.7 |
|  | C_1_ | 1757 | 20.3 |
| Cobm-SAH | C_0_ | 7228 | 60.5 |
|  | *C_1_ | 1711 | 39.5 |

*Indicates the representative conformation for the corresponding system

**Table S5.** Comparison of the secondary structure content of *Cp*-CobM determined by MD simulations and CD spectroscopy.

|  | **MD simulations** | | | **CD spectroscopy** | | |
| --- | --- | --- | --- | --- | --- | --- |
| **System** | α | β | random | α | β | random |
| **CobM** | 38.4% | 28.4% | 33.2% | 42% | 26% | 32% |
| **CobM-SAM** | 35.8% | 28.7% | 35.5% | 38% | 29% | 33% |
| **CobM-SAH** | 38.3% | 28.5% | 33.2% | - | - | - |

**Supplemental Video 1**. Based on the MD simulation results of *Cp*-CobM, *Cp*-CobM-SAM and *Cp*-CobM-SAH a video demonstrate the conformational changes in the region surrounding the SAM/SAH binding region dependent on the cofactor binding and its re-orientation after the CH_3_ group transfer to precorrin-4. The conformation changes effecting the SAM/SAH pocket size as described in supplemental Fig. S10 and this movements are also observed in the video. The molecular movements depending on the cofactor binding and may increase the accessibility of the precorrin-4 binding region.
